# Fermented fruit waste extract-loaded sodium alginate beads for the sustainable management of *Drosophila melanogaster* (Diptera: Drosophilidae)

**DOI:** 10.3389/finsc.2026.1933666

**Published:** 2026-09-28

**Authors:** Kannan Swathy, Perumal Vivekanandhan, Patcharin Krutmuang

**Affiliations:** 1 Department of Entomology and Plant Pathology, Faculty of Agriculture, Chiang Mai University, Chiang Mai, Thailand; 2 Office of Research Administration, Chiang Mai University, Chiang Mai, Thailand; 3 Center of Omics for High-Value Agriculture, Faculty of Agriculture, Chiang Mai University, Chiang Mai, Thailand

**Keywords:** 2-Propenoic acid, attractant, controlled-release formulation, drosophila melanogaster, fermented juice, fruit pest control, fruit waste, 6-methylheptyl ester

## Abstract

**Introduction:**

This study developed an eco-friendly attractant from fermented fruit waste juices for *Drosophila melanogaster* (Diptera: Drosophilidae), a model dipteran species, aligning with Sustainable Development Goals (SDGs) SDG 2 (Zero Hunger), 12 (Responsible Consumption and Production) and 15 (Life on Land) through sustainable waste valorization and reduced reliance on synthetic pesticides.

**Methods:**

Multi-choice bioassays were conducted to evaluate the attraction of *D. melanogaster* to fruit waste juices subjected to different fermentation durations. The microorganisms present in the most effective fermented banana juice were identified using morphological and molecular approaches. Gas chromatography–mass spectrometry (GC–MS) was used to characterize the volatile organic compound (VOC) profiles of fresh and fermented juices. To develop a greenhouse-deployable formulation, sodium alginate–carboxymethyl cellulose (CMC) beads loaded with fresh and fermented banana juice were evaluated under greenhouse conditions. Molecular docking analysis was performed against the odorant-binding protein dmelOBP28a (PDB ID: 6QQ4) to assess the predicted binding interactions of selected VOCs with the target protein.

**Results:**

Fermentation duration significantly enhanced insect attraction across all tested substrates, with banana juice (*Musa acuminata*) fermented for seven days eliciting the highest attraction rate (34.07%; F_(5,12)_ = 121.331; p < 0.001). The microorganisms present in the more effective fermented banana juice were identified as *Saccharomyces cerevisiae* (PX986379.1), *Lactiplantibacillus* plantarum (PX986372.1), and *Acetobacter aceti* (PX986373.1). GC–MS analysis showed that fermentation substantially diversified the VOC profile, yielding elevated levels of D-limonene, phenolic derivatives, esters, and organic acids that correlated with increased attraction. Under greenhouse conditions, sodium alginate–CMC beads loaded with seven-day fermented juice achieved a higher attraction rate (82.96%; F(2,6) = 358.183; p < 0.001) than those loaded with fresh juice, confirming the effectiveness of the controlled-release delivery system. Molecular docking analysis indicated that 2-Propenoic acid, 6-methylheptyl ester and D-limonene exhibited the strongest predicted binding affinities to dmelOBP28a, with docking scores of −6.7 and −6.5 kcal/mol, respectively.

**Discussion:**

These findings demonstrate that fermented fruit waste, particularly banana waste, can be developed into an affordable and environmentally friendly fruit fly attractant by combining microbial fermentation, chemical ecology, and controlled-release formulation techniques. The favorable predicted interactions of 2-Propenoic acid, 6-methylheptyl ester and D-limonene with dmelOBP28a suggest that these compounds may contribute to odor-mediated attraction in *D. melanogaster*. However, the present study did not directly evaluate reductions in fruit infestation or postharvest fruit losses. Further field and postharvest studies are therefore needed to determine the potential of this approach for reducing fruit damage and associated losses.

## 1 Introduction

*Drosophila melanogaster* (Meigen) (Diptera: Drosophilidae) is widely recognized as a premier genetic and biological model organism because of its well-characterized genome, short generation time, and extensive experimental utility (1). Beyond its importance in laboratory research, *D. melanogaster* is a cosmopolitan, fruit-associated drosophilid that commonly exploits ripe, overripe, damaged, fermenting, and decomposing fruits and other plant substrates. Commonly known as the vinegar fly or common fruit fly, it is frequently encountered in vineyards, fruit markets, food-processing facilities, and other environments where fermenting organic materials occur. Adults are strongly attracted to fermentation-associated odors, particularly volatile compounds produced through microbial activity, which serve as important cues for locating suitable feeding and reproductive substrates. In vineyard environments, *D. melanogaster* and *D. simulans* may constitute a substantial proportion of the drosophilid community, with abundance increasing as fruit ripens and harvesting progresses. Under favorable summer conditions, vinegar flies can complete development in approximately 7–8 days, and females may produce several hundred eggs during their adult lifespan, allowing populations to increase rapidly when suitable breeding substrates are continuously available (2).

The pest status of *D. melanogaster* differs substantially from that of *D. suzukii* (Matsumura), an economically important invasive fruit pest. *D. melanogaster* primarily exploits fruit that has already undergone ripening, physical damage, fermentation, or microbial deterioration, whereas females of *D. suzukii* possess a strongly sclerotized and serrated ovipositor that enables them to penetrate the skin of intact, ripening soft-skinned fruits and deposit eggs beneath the fruit surface. Consequently, *D. suzukii* can directly damage commercially marketable fruit before substantial external deterioration occurs. The agricultural importance of *D. melanogaster* is instead more closely associated with damaged and fermenting fruit, postharvest deterioration, contamination, and interactions with fruit-spoilage microorganisms (1–3). In grape production, drosophilid flies can also contribute to the epidemiology of grape sour rot by facilitating microbial transmission and creating conditions favorable for disease development, although they are not considered the sole cause of this disease complex (4, 5). Thus, monitoring and management of vinegar fly populations remain important for reducing contamination and deterioration in susceptible production systems.

Management of *D. melanogaster* and other vinegar flies commonly relies on sanitation, removal of overripe and damaged fruit, elimination of fermenting breeding substrates, exclusion, trapping, and, when appropriate, insecticide applications. However, repeated dependence on chemical insecticides raises concerns regarding resistance development, environmental contamination, residues, and effects on beneficial and non-target organisms (6–9). Chemical control may also be insufficient where abundant fermenting substrates continuously provide feeding and breeding resources. These limitations have increased interest in attractant-based monitoring and mass-trapping strategies that exploit the natural olfactory behavior of fruit flies and may reduce reliance on insecticides. Olfaction plays a central role in the ecology of *D. melanogaster*. Adults use volatile organic compounds (VOCs) to locate food sources, suitable substrates, and potential oviposition sites. Fermentation-associated odors are particularly important because microbial fermentation produces complex mixtures of alcohols, esters, acids, aldehydes, ketones, and other volatile compounds. Yeasts are especially important because they represent a major nutritional resource for *Drosophila* species and are closely associated with fermenting fruits. Consequently, flies have evolved olfactory mechanisms for detecting volatile cues associated with yeast and fermentation (10–12). Thus, attraction to fermentation odors reflects interactions among fruit-derived volatiles, microbial metabolites, and the nutritional characteristics of the substrate.

The chemical profile of fermentation-derived odors varies with fruit substrate, microbial community, temperature, oxygen availability, pH, moisture, and fermentation duration. Microorganisms metabolize sugars and other nutrients to produce ethanol, organic acids, esters, higher alcohols, and other volatile compounds, thereby altering the odor profile over time. For example, *Saccharomyces cerevisiae* produces ethanol and diverse volatile metabolites during alcoholic fermentation, whereas lactic acid bacteria and acetic acid bacteria contribute to acidification and the production or transformation of organic acids and other metabolites (13). Because the composition and abundance of these compounds depend on microbial and environmental conditions, relationships between fermentation chemistry and insect attraction should be evaluated experimentally rather than inferred solely from individual compounds (14, 15). Fruit-processing residues and discarded fruits represent promising resources for developing low-cost, fermentation-based attractants. Banana, pineapple, mango, guava, and papaya wastes contain carbohydrates and other nutrients that can support microbial growth and fermentation. Their conversion into attractants could simultaneously address fruit-waste management and the need for affordable pest-monitoring or control technologies, consistent with waste valorization and circular bioeconomy principles (16, 17). However, attractant performance may differ among fruit substrates because differences in sugar composition, acidity, native microbial communities, and volatile precursors can influence fermentation profiles.

Characterization of fermentation-derived VOCs can help explain differences in attractant performance among substrates and fermentation periods. GC–MS provides a useful platform for profiling volatile and semi-volatile compounds in fermented fruit materials. Alcohols, esters, organic acids, terpenoids, aldehydes, ketones, and other constituents contribute to fermented-fruit odor profiles and may influence insect behavior individually or through interactions. For example, D-limonene is commonly associated with citrus- and fruit-derived volatile profiles and has demonstrated biological activity in several insect systems (18, 19). Fermentation-derived esters, including ethyl acetate and related compounds, can also contribute characteristic fruity odors and potentially influence drosophilid olfactory behavior (20, 21). Nevertheless, detection of a compound by GC–MS does not establish its function as an attractant; behavioral assays are therefore required to evaluate the biological relevance of candidate compounds.

The detection of volatile cues involves a specialized olfactory system comprising odorant receptors, ionotropic receptors, odorant-binding proteins (OBPs), and associated sensory neurons. OBPs are soluble proteins within the sensillar lymph that bind and transport hydrophobic odorant molecules to olfactory receptors, facilitating the initial recognition of chemical signals. Molecular docking provides a computational approach for predicting ligand-binding poses and estimating relative binding affinities within defined protein-binding sites. Although docking scores cannot confirm behavioral attraction or fully elucidate odorant-recognition mechanisms, they can provide hypotheses regarding potential interactions between volatile compounds and olfactory proteins (22–26). In the present study, molecular docking was performed using the odorant-binding protein dmelOBP28a from *D. melanogaster* (PDB ID: 6QQ4). The crystal structure of dmelOBP28a provides a model for investigating potential interactions between candidate odor-active compounds and an insect OBP. Previous structural and functional studies have demonstrated that OBP28a participates in the detection and modulation of responses to specific odorants, providing a basis for evaluating whether fermentation-associated VOCs can interact favorably with its binding pocket. The docking results were interpreted as predictive evidence of potential ligand–OBP interactions rather than direct evidence of attractant activity; behavioral assays remain necessary to establish biological relevance.

Despite increasing interest in fermentation-derived insect attractants, important knowledge gaps remain. Previous studies have often focused on individual fruit substrates, specific synthetic compounds, or limited fermentation periods, whereas comparatively few studies have systematically compared multiple fruit wastes across different fermentation durations while integrating microbial characterization, VOC profiling, and insect behavioral responses. Consequently, the relationships among fermentation duration, microbial activity, VOC composition, and attractant efficacy remain insufficiently characterized. Accordingly, the present study aimed to develop and evaluate a sustainable fruit-waste-derived attractant system for *D. melanogaster*. Fermented wastes from banana, pineapple, mango, guava, and papaya were evaluated at different fermentation periods to determine their effects on adult fruit-fly attraction. The study further characterized the microorganisms associated with fermentation and profiled volatile compounds using GC–MS. Selected fermentation-associated VOCs were subsequently evaluated by molecular docking against dmelOBP28a to investigate their potential interactions with an olfactory binding protein. The most promising fermented fruit attractant was then incorporated into biodegradable sodium alginate–carboxymethyl cellulose (CMC) beads and evaluated under greenhouse conditions. This sequential workflow integrated fermentation biology, microbial characterization, chemical profiling, behavioral screening, molecular docking, and controlled-release formulation to evaluate the potential of fruit waste as a low-cost and environmentally sustainable attractant for *D. melanogaster* and to advance waste-valorization approaches for insect pest management.

## 2 Materials and methods

### 2.1 Collection of fruit waste

Five different types of fruit waste, namely banana (*Musa acuminata*, AAA Group, ‘Kluai Hom Thong’), pineapple (*Ananas comosus* ‘Pattavia’), mango (*Mangifera indica* ‘Nam Dok Mai’), guava (*Psidium guajava* ‘Farang Kimju’), and papaya (*Carica papaya* ‘Khaek Dam’), were collected from a local market in Chiang Mai, Thailand. The collected biomass was manually separated by fruit type and washed three times with distilled water to remove surface dirt, dust, and adherent microbial contaminants.

### 2.2 Preparation of fruit juice with varied fermentation periods

Fruit wastes from banana (*M. acuminata* AAA Group ‘Kluai Hom Thong’), pineapple (*A. comosus* ‘Pattavia’), mango (*M. indica* ‘Nam Dok Mai’), guava (*P. guajava* ‘Farang Kimju’), and papaya (*C. papaya* ‘Khaek Dam’) were used as fermentation substrates. The fruit wastes were cut into approximately 2–3 cm pieces using a sterile stainless-steel knife. For each fruit type, 500 g of freshly prepared fruit waste was mixed with 500 mL of sterile deionized water at a 1:1 (w/v) ratio and homogenized using an electric blender under aseptic conditions. The resulting slurry was first filtered through two layers of sterile muslin cloth and subsequently through Whatman No. 1 filter paper to obtain a clarified fruit extract.

The filtered extracts were transferred into sterile amber glass containers and subjected to spontaneous fermentation by the indigenous microorganisms naturally associated with the fruit waste. The containers were loosely capped to allow the release of fermentation gases while minimizing external contamination and were incubated in the dark at 25 ± 2 °C. Fermentation samples were aseptically collected at 0, 1, 3, 5, and 7 days of fermentation. The day-0 sample represented the freshly prepared, unfermented fruit extract and served as the baseline treatment. Samples collected at each fermentation period were subsequently used in the fruit-fly attraction bioassays. For each fruit type, three independent fermentation batches were prepared under identical conditions and treated as biological replicates (n = 3). All preparation and fermentation procedures were performed using aseptic techniques to minimize unintended external microbial contamination.

### 2.3 Insect culturing and maintanance

A laboratory colonies of *D. melanogaster* was established by collecting fruits infested by *D. melanogaster* in the field and maintained for several generations under controlled laboratory conditions to produce standardized insects for behavioral studies. The flies were kept in ventilated cages within a climate-controlled chamber set at 25 ± 2 °C, with 65–75% relative humidity, and a 12:12 h light-dark cycle, which are optimal conditions for their development and reproduction (27). The insects were provided with an artificial diet consisting of cornmeal, yeast extract, sucrose, and agar, which was refreshed every 3 to 4 d. All experimental protocols involving *D. melanogaster* adults were reviewed and approved by the Animal Care and Use Committee at the Faculty of Agriculture, Chiang Mai University, Thailand (Approval No. AG04001-2569).

### 2.4 Insect bioassays and selection

#### 2.4.1 Multiple-choice insect attractant bioassay

The chemotactic response of *D. melanogaster* was evaluated using a multiple-choice insect attraction bioassay (**Figure 1**). Fermented fruit juices from banana (*M. acuminata* AAA Group ‘Kluai Hom Thong’), pineapple (*A. comosus* ‘Pattavia’), mango (*M. indica* ‘Nam Dok Mai’), guava (*P. guajava* ‘Farang Kimju’), and papaya (*C. papaya* ‘Khaek Dam’) were prepared individually at different fermentation periods (0, 1, 3, 5, and 7 days) and compared against sterile deionized water, which served as the negative control. The bioassay was conducted inside a screened cage (120 × 60 × 90 cm) under the controlled laboratory conditions described above. For each treatment, 500 mL of fermented fruit juice was placed in a uniform plastic attractant container. Treatment and control containers were arranged evenly in a circular pattern within the cage, with their positions randomized between replicates to minimize positional bias.

**Figure 1:**
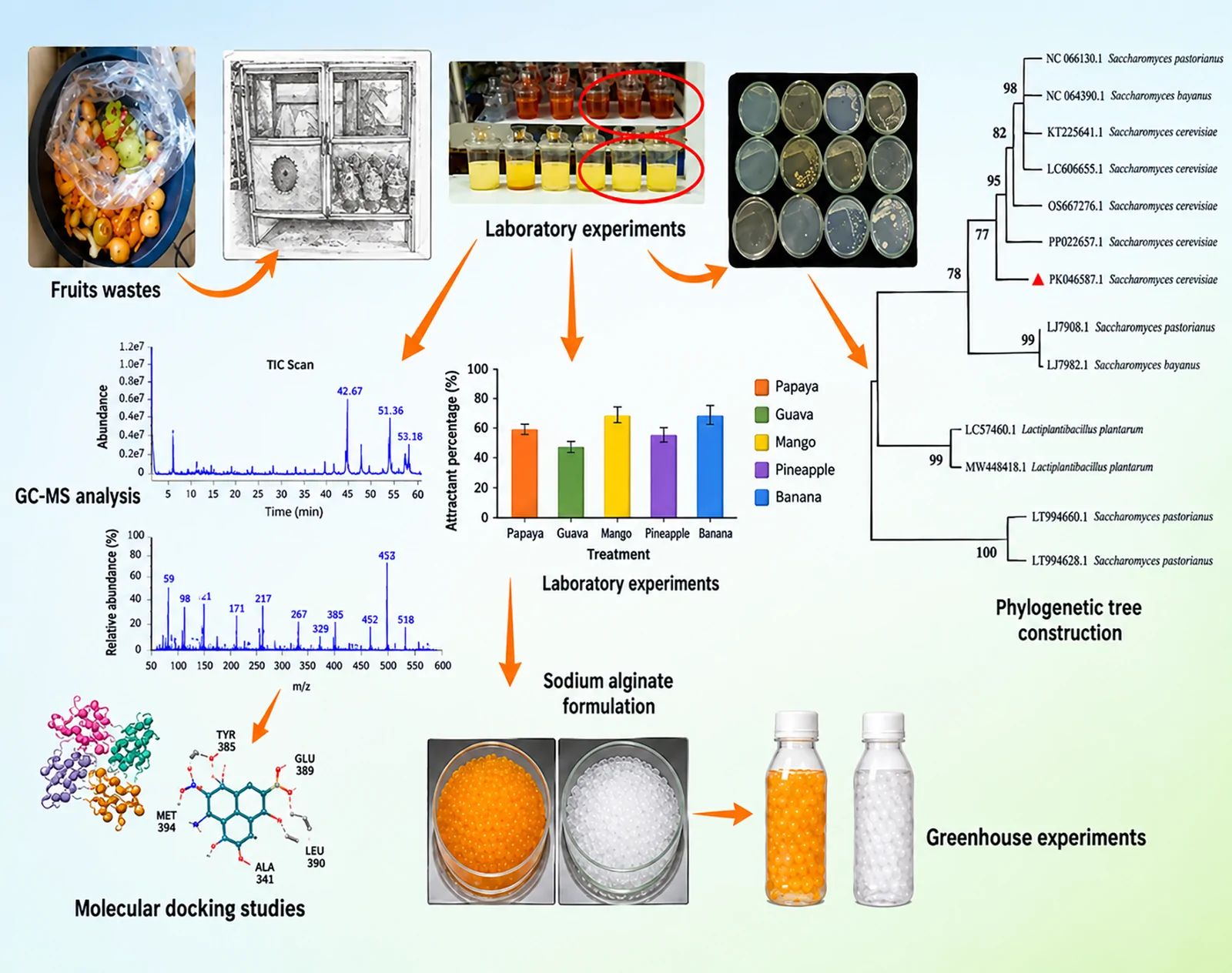
Overview of the experimental design and analytical workflow used for the development and evaluation of fruit waste-derived sodium alginate formulations aimed at attracting fruit flies.

Adult *D. melanogaster*, seven days post-emergence and of mixed sex, were used for the attraction assays. Each fruit juice was evaluated independently using the same experimental design. For each fruit juice, five treatment conditions corresponding to different fermentation periods were evaluated along with one unfermented/control treatment, resulting in a total of six treatment groups. Each treatment group consisted of three biological replicates, with 15 adult insects per replicate. Thus, 45 insects were used per treatment group and 270 insects were used in total for each fruit juice experiment (6 treatment groups × 3 replicates × 15 insects). The same experimental procedure and number of insects were used for each of the other fruit juices evaluated in the study. After release, the insects were allowed to move freely toward the attractant sources for 24 h under controlled conditions (25 ± 2 °C, 65 ± 5% relative humidity, and a 12:12 h light–dark cycle). At the end of the exposure period, the number of insects captured in each treatment container was recorded. The fruit-fly attraction % was calculated to quantify insect preference for each attractant relative to the control, using the following formula:


$$\text{Attraction }(\%)\;=\frac{\text{Number attracted to each choice​}}{\text{Total insects released}}\times 100$$

The treatment that exhibited the highest attraction response among the tested fruit types and fermentation periods was considered the most promising formulation and was selected for subsequent laboratory optimization and greenhouse evaluation. This sequential screening approach ensured that the greenhouse experiment was based on the best-performing formulation identified under controlled laboratory conditions.

#### 2.4.2 Selection of potent fermented fruit juices

The most effective fermented fruit juice was selected based on the attraction response of adult *D. melanogaster* to fruit juices fermented for different durations (0, 1, 3, 5, and 7 days), with sterile distilled water serving as the control. Fruit-fly attraction was evaluated after 24 h of exposure based on the percentage of adults attracted to each treatment. The fermented fruit juice that produced the highest statistically significant attraction response was selected for subsequent analyses. The selected fermented fruit juice was subsequently subjected to microbial isolation and molecular identification to characterize the microorganisms associated with the fermentation process and to identify dominant microbial taxa potentially involved in the production of fermentation-derived metabolites.

In parallel, volatile organic compounds (VOCs) present in the selected fermented juice were characterized using gas chromatography–mass spectrometry (GC–MS) to identify major volatile constituents potentially associated with fruit-fly attraction. To further investigate the potential molecular basis of attraction, molecular docking analyses were performed to predict the interactions between selected fermentation-associated VOCs and the odorant-binding protein dmelOBP28a of *D. melanogaster*. The docking analysis was used to provide mechanistic insights into the potential interactions between candidate volatile compounds and the insect olfactory system and was not considered direct evidence of behavioral attraction. Finally, the selected fermented fruit juice was incorporated into a sodium alginate–carboxymethyl cellulose (CMC) bead formulation for controlled release, and its attractant performance was subsequently evaluated under greenhouse conditions to assess its effectiveness under greenhouse conditions.

### 2.5 Microbial isolation and identification

#### 2.5.1 Isolation of microbes from potent fermented juices

The 7-day-fermented banana waste juice prepared from *M. acuminata* AAA Group ‘Kluai Hom Thong’, which exhibited the highest attraction response toward adult *D. melanogaster*, was selected for microbial isolation. Samples of the fermented juice were collected aseptically and serially diluted (10^-1^–10^-6^) using sterile 0.85% (w/v) saline solution. Aliquots (100 µL) from appropriate dilutions were spread onto the respective culture media for the isolation of yeast and bacterial microorganisms. Yeasts were cultured on Yeast Extract–Peptone–Dextrose (YPD) agar, whereas bacteria were cultured on Nutrient Agar (NA). The plates were incubated aerobically at 28–30 °C for 48–72 h. Morphologically distinct colonies showing consistent growth were selected for purification. Representative yeast and bacterial colonies were repeatedly subcultured onto fresh respective media until pure cultures were obtained. Three representative microbial isolates were selected for subsequent morphological and molecular characterization based on colony morphology, growth characteristics, and their occurrence among the recovered isolates. The purified isolates were maintained on agar slants at 4 °C for short-term use and preserved in 25% (v/v) glycerol at −80 °C for long-term storage.

#### 2.5.2 Morphological characterization

The purified microbial isolates were initially characterized based on colony morphology and microscopic characteristics before molecular identification. Colony morphology was assessed after incubation on the respective culture media, and characteristics including colony size, shape, color, pigmentation, opacity, surface texture, elevation, and margin were recorded. Microscopic observations were performed using a light microscope at 1,000× magnification under oil immersion.

Yeast isolates were stained with methylene blue and examined for cellular morphology, budding pattern, and cell viability. Bacterial isolates were subjected to Gram staining to determine their Gram reaction, cellular morphology, and arrangement. Bacterial motility, when required, was assessed using the hanging-drop method. These phenotypic and microscopic characteristics were used for preliminary characterization of the isolates and to complement subsequent molecular identification (28).

#### 2.5.3 Genomic DNA extraction

Genomic DNA was extracted from purified microbial cultures grown overnight in Yeast Extract–Peptone–Dextrose (YPD) broth for yeast isolates and Nutrient Broth (NB) for bacterial isolates at 28–30 °C under aerobic conditions. The cultures were harvested by centrifugation at 8,000 × g for 5 min, and the resulting cell pellets were washed twice with sterile phosphate-buffered saline (PBS; pH 7.2). For yeast isolates, cell disruption was facilitated by treatment with lyticase at 37 °C for 30 min, whereas bacterial cells were treated with lysozyme at 37 °C for 30 min. Genomic DNA was subsequently extracted using a commercial microbial DNA extraction kit according to the manufacturer’s instructions (QIAGEN,Germany). DNA concentration and purity were assessed spectrophotometrically by measuring absorbance at 260 and 280 nm, and DNA purity was evaluated based on the A260/A280 ratio. DNA integrity was further assessed by electrophoresis on a 1% (w/v) agarose gel containing an appropriate nucleic acid stain and visualized using a gel documentation system. Extracted DNA was stored at −20 °C until PCR amplification.

#### 2.5.4 PCR Amplification

Microbial isolates were identified at the molecular level by amplifying taxonomically informative genetic markers using polymerase chain reaction (PCR), followed by DNA sequencing. For yeast identification, the internal transcribed spacer (ITS) region was targeted, while bacterial isolates were identified by amplifying the 16S rRNA gene. Both markers are widely recognized as standard molecular barcodes for microbial taxonomy (29).

#### 2.5.5 Amplification of Yeast ITS Region

Yeast isolates were identified by amplification of the internal transcribed spacer (ITS1–5.8S–ITS2) region of the ribosomal DNA using the universal primers ITS1 (5′-TCCGTAGGTGAACCTGCGG-3′) and ITS4 (5′-TCCTCCGCTTATTGATATGC-3′) (29). PCR was performed in a total reaction volume of 25 µL containing genomic DNA template, 1× PCR buffer, MgCl_2_, dNTPs, 0.4 µM each of the forward and reverse primers, Taq DNA polymerase, and nuclease-free water. PCR amplification consisted of an initial denaturation at 95 °C for 5 min, followed by 35 cycles of denaturation at 95 °C for 30 s, annealing at 55 °C for 30 s, and extension at 72 °C for 1 min, followed by a final extension at 72 °C for 7 min. The amplified products were analyzed by agarose gel electrophoresis to confirm successful amplification before purification and sequencing. The ITS region was selected because it is widely used as a molecular marker for fungal and yeast identification owing to its relatively high interspecific variability and conserved flanking regions (29).

#### 2.5.6 Amplification of bacterial 16s rRNA gene

Bacterial isolates were identified by amplification of the 16S rRNA gene using the universal bacterial primers 27F (5′-AGAGTTTGATCMTGGCTCAG-3′) and 1492R (5′-TACGGYTACCTTGTTACGACTT-3′). PCR reactions were prepared in a total volume of 25 µL using the appropriate PCR buffer, MgCl_2_, dNTPs, forward and reverse primers, Taq DNA polymerase, genomic DNA template, and nuclease-free water. PCR amplification was performed with an initial denaturation at 95 °C for 5 min, followed by 35 cycles of denaturation at 95 °C for 30 s, annealing at 55 °C for 30 s, and extension at 72 °C for 90 s, followed by a final extension at 72 °C for 10 min. The amplified products were electrophoresed on a 1.5% (w/v) agarose gel prepared in 1× TAE buffer and visualized using an appropriate nucleic acid stain. PCR products of the expected size were purified using a commercial PCR purification kit according to the manufacturer’s instructions and subjected to Sanger sequencing.

#### 2.5.7 Phylogenetic tree construction

The purified PCR products were sequenced bidirectionally using the Sanger sequencing method. Forward and reverse chromatograms were inspected for sequence quality, and high-quality reads were assembled to generate consensus sequences. Low-quality regions and ambiguous nucleotide positions were removed before downstream analysis. The resulting consensus sequences were compared with reference sequences available in the NCBI GenBank database using BLASTn for preliminary taxonomic identification (30). Closely related reference sequences were retrieved from GenBank based on BLAST results and relevant taxonomic information. Multiple sequence alignments were performed using the ClustalW algorithm (31). Phylogenetic relationships among the isolates and reference sequences were inferred using the Neighbor-Joining method implemented in MEGA version 11 (32). Branch support was assessed using 1,000 bootstrap replicates. The resulting phylogenetic trees were visualized and edited using MEGA 11. The sequences generated in this study were deposited in the NCBI GenBank database, and the corresponding accession numbers are provided in the manuscript.

### 2.6 Extraction of crude chemical constituents

Volatile and semi-volatile compounds from fresh (0-day) and fermented (7-day) banana juice were extracted using liquid–liquid extraction with analytical-grade ethyl acetate and subsequently analyzed by gas chromatography–mass spectrometry (GC–MS). Ripened banana waste of *M. acuminata* AAA Group ‘Kluai Hom Thong’ was washed thoroughly with distilled water, aseptically homogenized, and filtered through sterile muslin cloth to obtain the fresh banana juice (0-day sample). A portion of the freshly prepared juice was subjected to spontaneous fermentation for 7 days under the same conditions described in Section 2.2 to obtain the fermented sample. Fresh and 7-day-fermented banana juice samples were centrifuged at 8,000 × g for 10 min at 4 °C to remove suspended solids and microbial cells. The resulting supernatants were transferred to clean separatory funnels and extracted with analytical-grade ethyl acetate at a 1:1 (v/v) sample-to-solvent ratio. The mixture was gently but thoroughly mixed for 10 min with intermittent venting to prevent excessive pressure buildup. After complete phase separation, the upper organic phase was carefully collected. The aqueous phase was re-extracted twice using the same procedure, and the organic fractions were pooled. The combined organic extracts were dried over anhydrous sodium sulfate (Na_2_SO_4_) to remove residual water and subsequently filtered through an appropriate solvent-resistant membrane filter. The dried extracts were concentrated under reduced pressure using a rotary evaporator at 35–40 °C. The concentrated extracts were transferred into amber GC–MS vials, tightly sealed, and stored at −20 °C until analysis. All extraction procedures were performed under conditions that minimized exposure to heat and light to reduce the loss or degradation of volatile compounds.

### 2.7 GC-MS analysis

Volatile and semi-volatile metabolites present in fresh (0-day) and fermented (7-day) banana waste juice extracts were charectrized using gas chromatography–mass spectrometry (GC–MS). The analysis was performed using a gas chromatograph coupled with a quadrupole mass spectrometer equipped with an electron ionization (EI) source. Metabolite separation was achieved using an HP-5MS capillary column (30 m × 0.25 mm i.d., 0.25 µm film thickness). Helium (99.999% purity) was used as the carrier gas at a constant flow rate of 1.0 mL min^-1^. The ethyl acetate extracts (1 µL) were injected in split mode (split ratio 10:1) with the injector temperature maintained at 250 °C. The oven temperature program was set as follows: initial temperature of 50 °C held for 3 min, increased to 150 °C at a rate of 5 °C min^-1^, followed by an increase to 280 °C at 8 °C min^-1^ and held for 10 min. The mass spectrometer was operated under EI conditions at 70 eV, with the ion source and quadrupole temperatures maintained at 230 °C and 150 °C, respectively. Mass spectra were acquired in full-scan mode over an m/z range of 40–600 with a solvent delay of 3 min. Chemical constituents identification was performed by comparing obtained mass spectra with reference spectra available in the NIST and Wiley libraries.

### 2.8 Formulation and applied testing

#### 2.8.1 Development of attractant formulations

Attractant formulations were prepared by encapsulating fresh (0-day) and 7 days old fermented fruit juice extracts in sodium alginate–carboxymethyl cellulose (CMC) hydrogel beads to develop controlled-release delivery systems (**Figure 2**). Sodium alginate was selected as the primary encapsulating polymer due to its ability to form hydrogels through calcium-mediated ionotropic gelation, while CMC was incorporated as a viscosity-modifying and structural stabilizing agent to improve bead integrity and regulate compound diffusion (33). A 2% (w/v) sodium alginate solution was prepared in distilled water with continuous stirring until complete dissolution. A separate 1% (w/v) CMC solution was prepared and combined with the alginate solution to obtain a homogeneous polymer matrix. Fresh or fermented fruit juice extracts were combined into the polymer blend under gentle stirring to ensure uniform delivery of bioactive attractant compounds. The resulting formulation was extruded dropwise through a sterile syringe into a 2% (w/v) calcium chloride (CaCl_2_) solution to induce ionic crosslinking and formation of spherical hydrogel beads (34). The formed beads were cured for 20–30 min, recovered by filtration, and washed with sterile distilled water to remove residual calcium ions. The beads were then air-dried under sterile conditions to remove surface moisture and stored in sealed containers at 4 °C until further bioassays.

**Figure 2:**
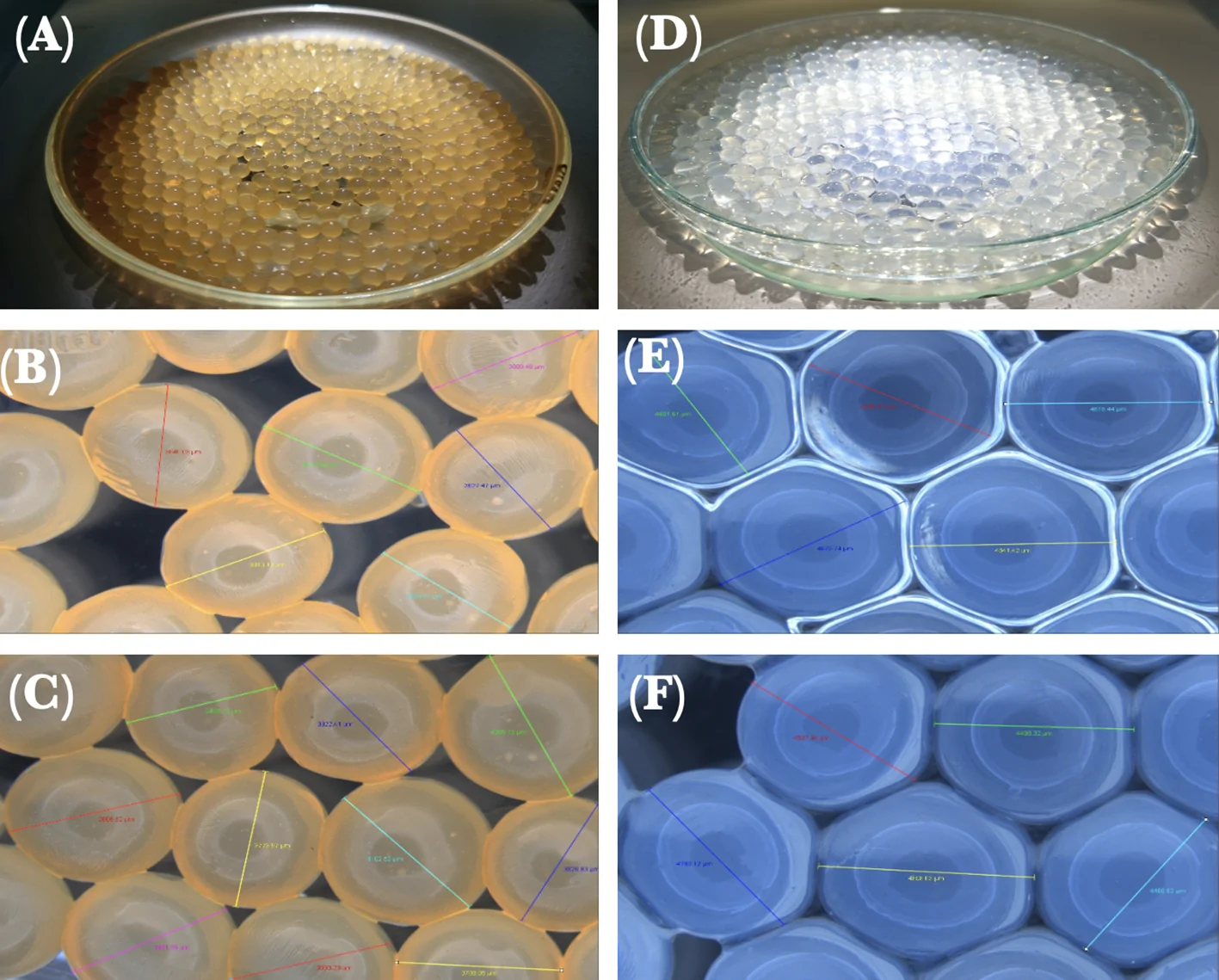
**(A–C)** Controlled-release sodium alginate–carboxymethyl cellulose (CMC) bead formulations loaded with 7 days old fermented banana fruit waste juice extract, showing an average bead size of 3792.32 µm. **(D–F)** Sodium alginate–CMC beads loaded with fresh banana fruit waste juice extract, exhibiting a larger average bead size of 4797.94 µm.

### 2.9 Greenhouse insect attractant bioassay

Following the laboratory multiple-choice screening, the fruit juice formulation that elicited the highest attractant response was selected for evaluation in a greenhouse setting. To validate the laboratory results under more realistic environmental conditions, the attractant efficacy of sodium alginate–CMC bead formulations containing fresh (0-day) and 7-day fermented fruit juice was assessed under controlled greenhouse conditions. Distilled water was used as the control. The greenhouse experiment was conducted in an insect-proof facility maintained at 30 ± 2 °C, 65–75% relative humidity, and a 12:12-hour light–dark photoperiod. Sodium alginate–CMC beads containing fresh fruit juice (0-day), selected 7-day fermented fruit juice, and distilled water were placed in standardized trap holders at equivalent positions within the experimental cages. The attractant formulations were exposed for 30 minutes before insect release to allow stabilization of volatile emissions. Mesh cages were used to contain the insects while permitting the dispersion of volatile compounds from the bead formulations. Seven-day-old, mixed-sex adult *D. melanogaster* were starved for 4 hours before release to standardize their feeding status and olfactory responsiveness (35). Fifteen adults were released from the center of each cage for each replicate. The number of adults attracted to each treatment was recorded after 24 hours. Each treatment was evaluated using three independent replicates, with 15 adults per replicate, resulting in 45 adults per treatment and 135 adults across the three treatment groups in each experimental set. The fruit fly attraction percentage was calculated based on the number of adults attracted to each treatment relative to the total number of adults released, using the following equation:


$$\text{Attraction }(\%)=\frac{\text{Number attracted to each choice​}}{\text{Total insects released}}\times 100$$

The greenhouse experiment was used as a validation step following laboratory screening, enabling a comparison between the selected fermented formulation and fresh fruit juice under controlled yet more environmentally representative conditions.

### 2.10 Computational and statistical validations

#### 2.10.1 Molecular docking analysis

Molecular docking analysis was performed to investigate the predicted interactions between selected volatile compounds identified by GC–MS and the odorant-binding protein dmelOBP28a of *D. melanogaster* (PDB ID: 6QQ4). The three-dimensional structure of dmelOBP28a was retrieved from the Protein Data Bank (PDB), and the chemical structures of selected volatile compounds, including 2-Propenoic acid, 6-methylheptyl ester (**Figure 3**), D-limonene (**Figure 4**), ethyl 2,3-epoxybutyrate (**Figure 5**), were obtained from the PubChem database. The selected ligand structures were energy-minimized using the MMFF94 force field before docking. The protein structure was prepared by removing crystallographic water molecules and non-essential heteroatoms or ligands, followed by the addition of polar hydrogen atoms and assignment of appropriate atomic charges. Docking simulations were subsequently performed using the CB-Dock2 web server, which employs the AutoDock Vina docking algorithm. Potential ligand-binding cavities were predicted automatically by the platform, and the selected ligands were docked into the predicted binding pockets.

**Figure 3:**
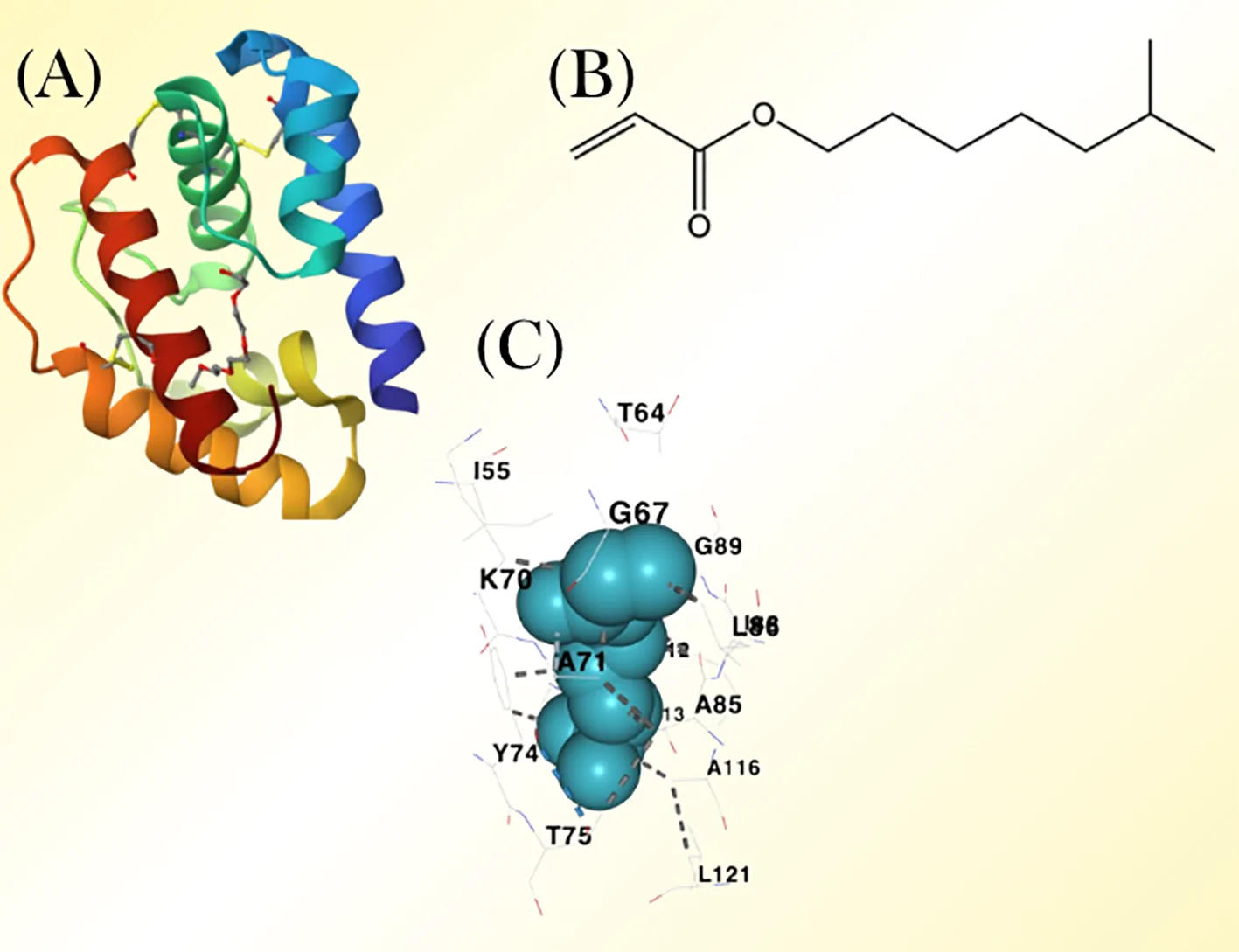
**(A)** Molecular docking interaction of odorant-binding protein dmelOBP28a from *D. melanogaster* (PDB ID: 6QQ4) with **(B)** 2-propenoic acid, 6-methylheptyl ester (PubChem CID: 165746). **(C)** ligand-binding pocket (C1) exhibited a docking score of −6.7 kcal/mol, the most favorable among five predicted pockets and corresponding to the largest cavity volume (1468 Å³), suggesting a stable and highly specific binding orientation within the protein’s principal hydrophobic cavity. Key interacting amino acid residues facilitating this complex, all located within Chain A, include Glu3, Ala6, Leu7, Leu10, Glu30, Met31, Lys34, Gln35, Arg46, Met50, Ile55, Leu62, Asp63, Thr64, Glu65, Ala66, Gly67, His68, Lys70, Ala71, Gln73, Tyr74, Thr75, Ala85, Leu86, Ile88, Gly89, Asp90, Glu106, Gly109, Thr110, Phe112, Arg113, Ala116, Leu121, and Leu122. These extensive intermolecular contacts, combining a broad hydrophobic surface (Leu7, Leu10, Met31, Met50, Ile55, Leu62, Ala66, Ala71, Ala85, Leu86, Ile88, Phe112, Leu121, Leu122) with polar/charged residues near the pocket entrance (Glu3, Glu30, Lys34, Gln35, Arg46, Asp63, Glu65, His68, Lys70, Asp90, Glu106, Arg113), underscore the strong predicted binding affinity and structural stability of the ligand within the odorant-binding pocket of dmelOBP28a.

**Figure 4:**
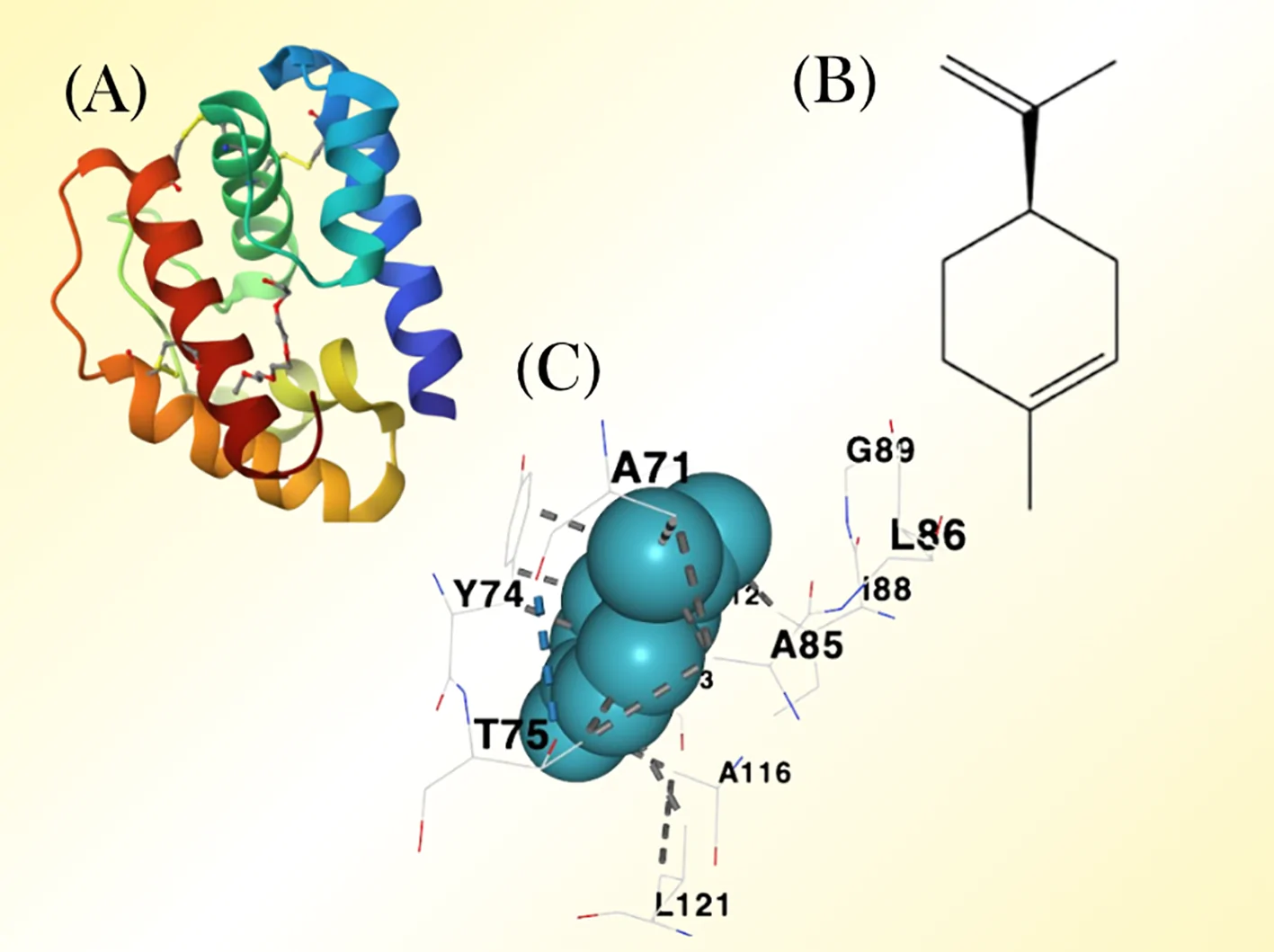
**(A)** Molecular docking interaction of odorant-binding protein dmelOBP28a from *D. melanogaster* (PDB ID: 6QQ4) with **(B)** D-limonene (PubChem CID: 440917). **(C)** ligand-binding pocket (C1) exhibited a docking score of −6.5 kcal/mol, the most favorable among five predicted pockets, corresponding to the largest cavity volume (1468 Å³) and suggesting a stable, specific binding orientation within the protein’s principal hydrophobic cavity. Key interacting amino acid residues facilitating this complex, all located within Chain A, include Glu3, Ala6, Leu7, Leu10, Met31, Lys34, Met50, Ile55, Thr64, Lys70, Ala71, Gln73, Tyr74, Thr75, Ala85, Leu86, Ile88, Gly89, Gly109, Phe112, Arg113, Ala116, Leu121, and Leu122. These intermolecular contacts, dominated by hydrophobic and aliphatic residues (Leu7, Leu10, Met31, Met50, Ile55, Ala71, Ala85, Leu86, Ile88, Phe112, Leu121, Leu122), underscore the favorable binding affinity and structural stability of D-limonene within the odorant-binding pocket of dmelOBP28a.

**Figure 5:**
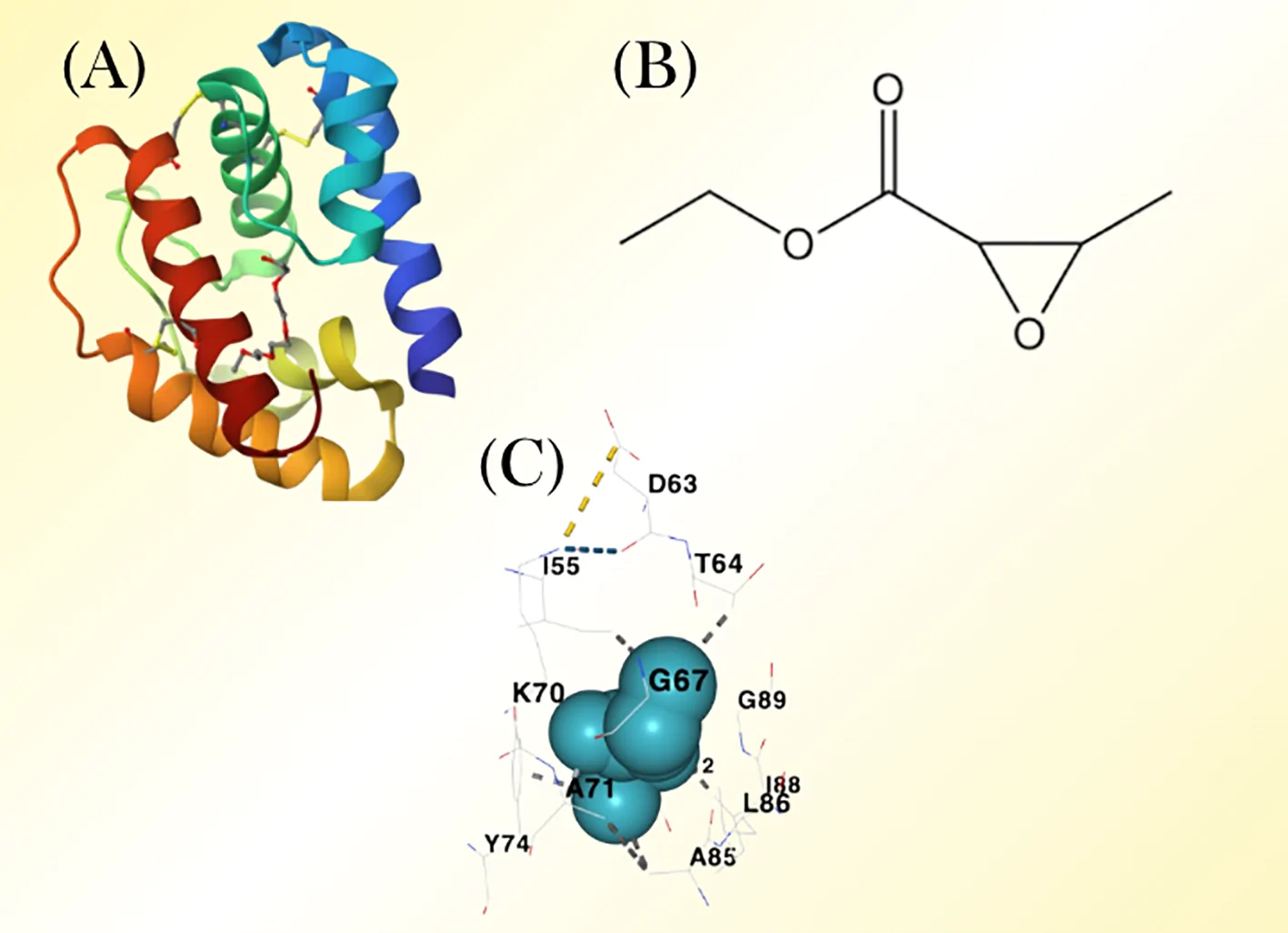
**(A)** Molecular docking interaction of odorant-binding protein dmelOBP28a from *D. melanogaster* (PDB ID: 6QQ4) with **(B)** ethyl 2,3-epoxybutyrate (PubChem CID: 29774). **(C)** ligand-binding pocket (C1) exhibited a docking score of −5.0 kcal/mol, the most favorable among five predicted pockets and corresponding to the largest cavity volume (1468 Å³), suggesting a stable and specific binding orientation within the protein’s principal hydrophobic cavity. Key interacting amino acid residues facilitating this complex, all located within Chain A, include Leu7, Leu10, Met31, Lys34, Gln35, Arg46, Val49, Met50, Ile55, Asp63, Thr64, Gly67, Lys70, Ala71, Tyr74, Thr75, Ala85, Leu86, Ile88, Gly89, Asp90, Glu106, Gly109, Thr110, Phe112, Arg113, Ala116, Leu121, and Leu122. These intermolecular contacts, combining hydrophobic residues (Leu7, Leu10, Met31, Val49, Met50, Ile55, Ala71, Ala85, Leu86, Ile88, Phe112, Leu121, Leu122) with polar/charged residues (Lys34, Gln35, Arg46, Asp63, Lys70, Asp90, Glu106, Arg113), underscore the favorable binding affinity and structural stability of ethyl 2,3-epoxybutyrate within the odorant-binding pocket of dmelOBP28a.

Docking poses were ranked according to the predicted Vina scoring values, expressed in kcal mol^-1^, with more negative scores indicating more favorable predicted ligand–protein interactions within the scoring framework. The top-ranked poses were selected for subsequent structural analysis. Protein–ligand interactions were visualized using Discovery Studio Visualizer and PyMOL, and the predicted interactions were examined with respect to hydrogen bonding, hydrophobic interactions, van der Waals contacts, and other non-covalent interactions. The amino acid residues forming interactions with the docked ligands were identified from the corresponding protein–ligand complexes. Where applicable, residues within the predicted binding pocket were compared among the different ligands to evaluate similarities and differences in their predicted interaction profiles. Comparative analysis of the docking scores and interaction patterns was used to assess the potential molecular interactions of the selected volatile compounds with dmelOBP28a and to generate hypotheses regarding their possible involvement in odorant recognition in *D. melanogaster*.

### 2.11 Statistical analysis

Data obtained from the laboratory and greenhouse attractant bioassays were expressed as fruit fly attraction (%), calculated as the number of *D. melanogaster* adults attracted to each treatment relative to the total number of insects released. The attraction percentage data were subjected to one-way analysis of variance (ANOVA) to determine whether significant differences existed among the tested attractant treatments. When a significant treatment effect was detected, treatment means were compared using Tukey’s honestly significant difference (HSD) *post hoc* test. Statistical significance was evaluated at a significance level of p < 0.05. All statistical analyses were performed using GraphPad Prism statistical software, version 9 (GraphPad Software, San Diego, CA, USA).

## 3 Results

### 3.1 Attraction response of *D. melanogaster*

Fermentation duration significantly affected the attractant activity of fruit-waste juice formulations against adult *D. melanogaster* (**Figure 6**). Across all fruit substrates evaluated, fermented juice treatments generally exhibited greater attraction than the corresponding fresh (0-day) treatments, indicating that fermentation enhanced the attractiveness of the fruit-waste-derived baits. Among the fruit wastes tested, banana (*M. acuminata* AAA Group ‘Kluai Hom Thong’) juice exhibited the highest attractant activity. Attraction increased progressively with fermentation duration and reached 34.07% after 7 days of fermentation (F_(5,12)_ = 121.331, *p* < 0.001), representing the strongest response among the fruit substrates evaluated (**Figure 6**). The enhanced attraction to fermented banana juice suggests that fermentation-associated biochemical changes increased the production and release of volatile compounds that may act as olfactory cues for *D. melanogaster*. Pineapple (*A. comosus* ‘Pattavia’) waste juice also showed a significant fermentation-dependent increase in attraction, reaching 26.30% after 7 days of fermentation (F_(5,12)_ = 131.861, *p* < 0.001; **Figure 6**).

**Figure 6:**
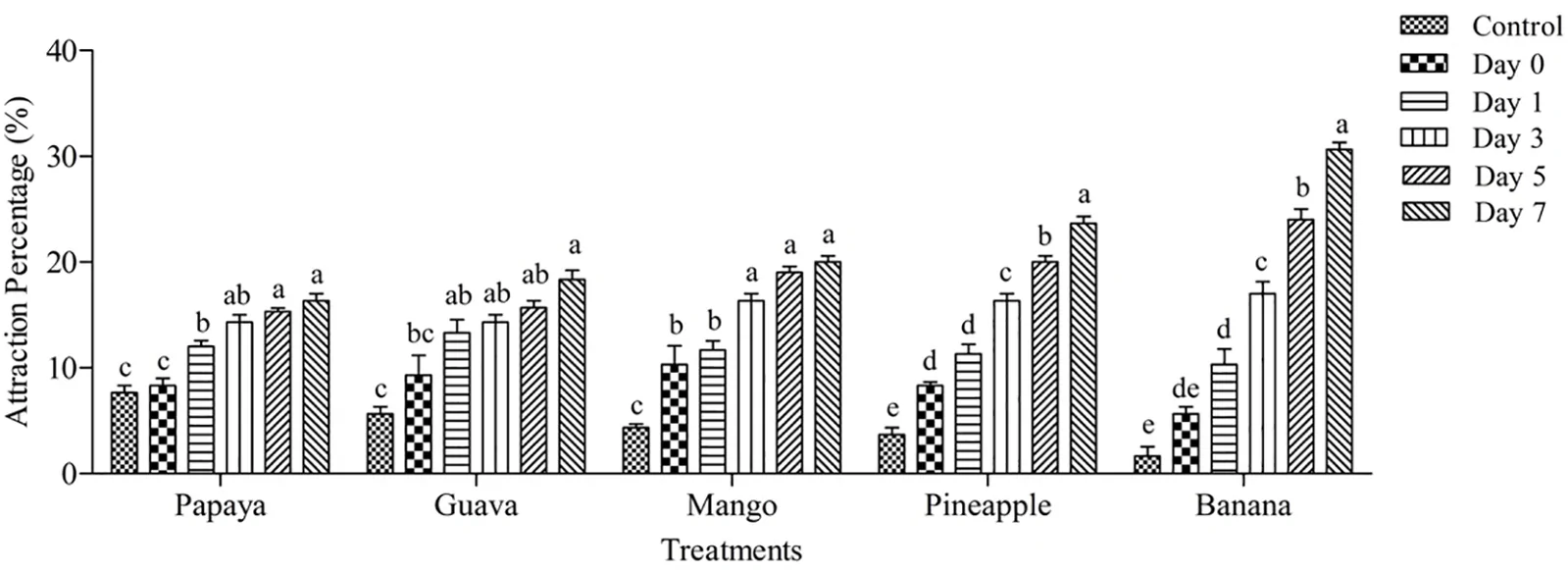
Laboratory attraction of *D. melanogaster* to various fruit waste-derived juices was tested. Treatments included a control and five fermentation periods (0 fresh juice, 1, 3, 5, and 7 days) for papaya, guava, mango, pineapple, and banana wastes.

Similarly, mango (*M. indica* ‘Nam Dok Mai’) waste juice exhibited significantly increased attraction following fermentation, reaching 22.22% after 7 days (F_(5,12)_ = 41.761, *p* < 0.001; **Figure 6**). Guava (*P. guajava* ‘Farang Kimju’) and papaya (*C. papaya* ‘Khaek Dam’) waste juices exhibited comparatively lower attractant activity. For guava, no significant difference was observed between the fresh (0-day) juice and the control; however, fermentation for 1–7 days significantly increased attraction, with the response reaching 20.37% after 7 days (F_(5,12)_ = 9.164, *p* < 0.001; **Figure 6**). Papaya showed a similar pattern, with the fresh juice producing no significant attraction relative to the control, whereas fermentation progressively increased attraction, reaching 18.15% after 7 days (F_(5,12)_ = 36.120, *p* < 0.001; **Figure 6**). Based on comparative laboratory screening, the 7-day fermented banana waste juice was selected as the most promising formulation because it elicited the highest attraction response (34.07%) among all fruit-waste treatments evaluated. This formulation was subsequently advanced to further formulation and greenhouse experiments to assess whether its attractant performance could be maintained under more environmentally representative conditions.

### 3.2 Selection of potent fermented fruit juices

Based on the screening results presented in **Figure 6**, the 7-day-fermented banana waste juice (*M. acuminata* AAA Group ‘Kluai Hom Thong’) exhibited the highest attractant activity against adult *D. melanogaster*. Attraction increased from 6.29% in the fresh (0-day) juice to 34.07% after 7 days of fermentation, with the 7-day treatment showing the highest attraction response among the tested fermentation periods. Therefore, the 7-day-fermented banana juice was selected for subsequent microbial isolation and identification, volatile and semi-volatile compound profiling by GC–MS, molecular docking analysis, and development of the sodium alginate–CMC bead formulation for greenhouse evaluation.

### 3.3 Morphological characterization

Morphological and microscopic characterization revealed distinct differences among the isolated microbial groups. The yeast isolate produced creamy, smooth, and convex colonies with entire margins. Microscopic examination showed oval-shaped cells with budding characteristics, indicating typical yeast-like morphology. Among the bacterial isolates, one exhibited rod-shaped, Gram-positive cells with smooth, opaque colonies, consistent with characteristics commonly observed in lactic acid bacteria. The other bacterial isolate showed Gram-negative, rod-shaped cells with colony morphology associated with acetic acid bacterial groups. Variations in motility were also observed among the bacterial isolates. These morphological and phenotypic characteristics supported the preliminary differentiation of the isolates into yeast, lactic acid bacteria, and acetic acid bacteria groups, which were further confirmed through molecular identification.

### 3.4 Molecular characterization

Molecular identification based on taxonomically relevant genetic markers confirmed the identities of the microbial isolates. The yeast isolate was identified through sequencing of the internal transcribed spacer (ITS) region, generating a high-quality sequence of the expected size (~550–650 bp), and was assigned as *Saccharomyces cerevisiae* (GenBank accession: PX986379.1) (**Figure 7**). The bacterial isolates were identified based on nearly full-length 16S rRNA gene sequences (~1,400–1,500 bp). Sequence analysis identified the isolates as *Lactiplantibacillus plantarum* (GenBank accession: PX986372.1) (**Figure 8**) and *Acetobacter aceti* (GenBank accession: PX986373.1) (**Figure 8**). BLASTn analysis showed 100% sequence similarity with corresponding reference sequences available in the NCBI GenBank database, supporting species-level identification. Phylogenetic analysis further confirmed the taxonomic placement of the isolates within their respective microbial groups. The identified microorganisms are commonly associated with fruit fermentation and may contribute to biochemical transformations through the production of organic acids, alcohols, and volatile metabolites, which could influence the chemical composition and biological activity of fermented fruit waste extracts.

**Figure 7:**
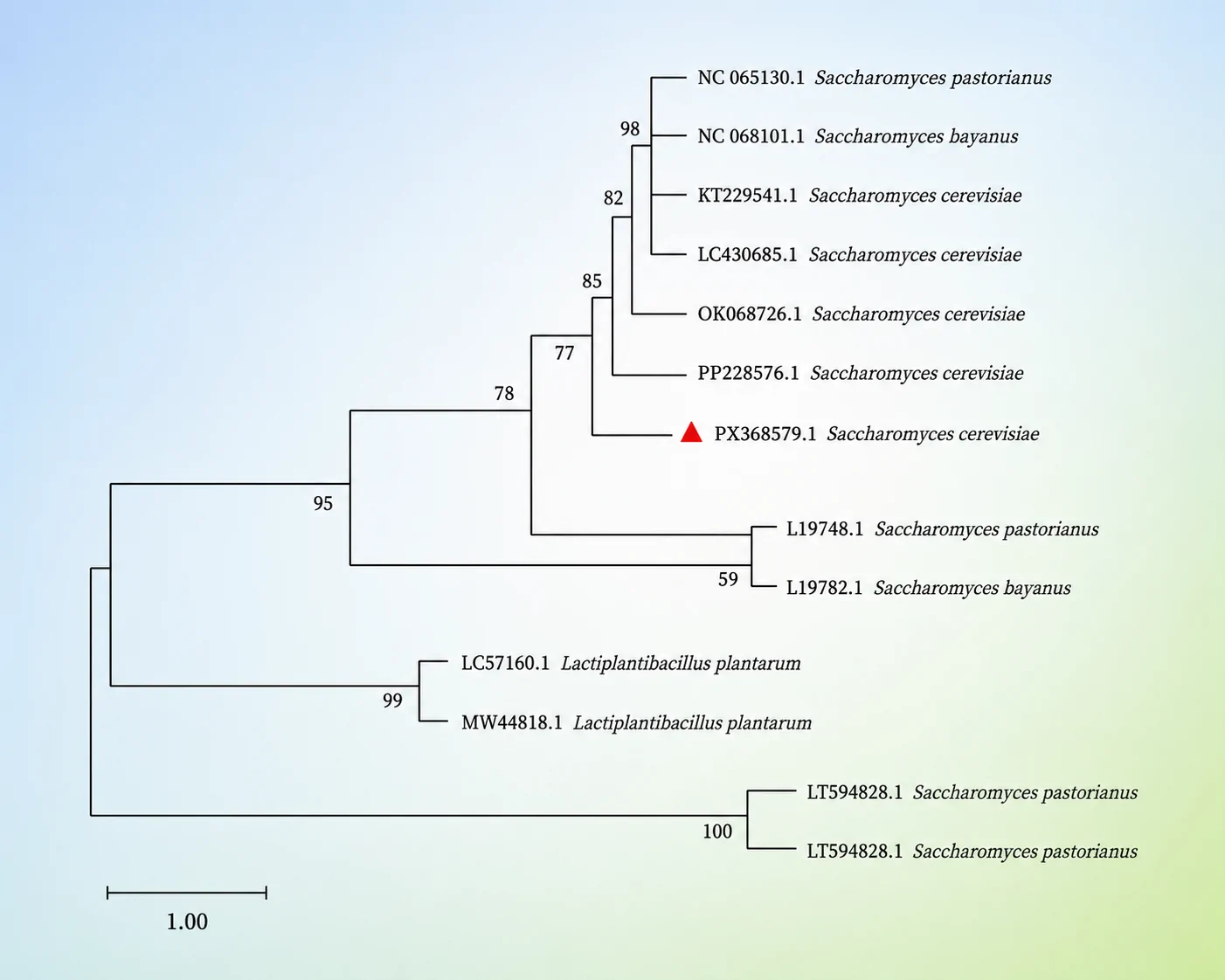
Molecular phylogenetic analysis was conducted on the yeast strain isolated from the most potent 7-day fermented banana juice. The evolutionary history was inferred using the Neighbor-Joining method. The phylogenetic tree illustrates the relationship between the query sequence (indicated by a red star) and closely related reference strains retrieved from the NCBI GenBank database. The isolate was identified as *S. cerevisiae* (GenBank accession: PX986379.1).

**Figure 8:**
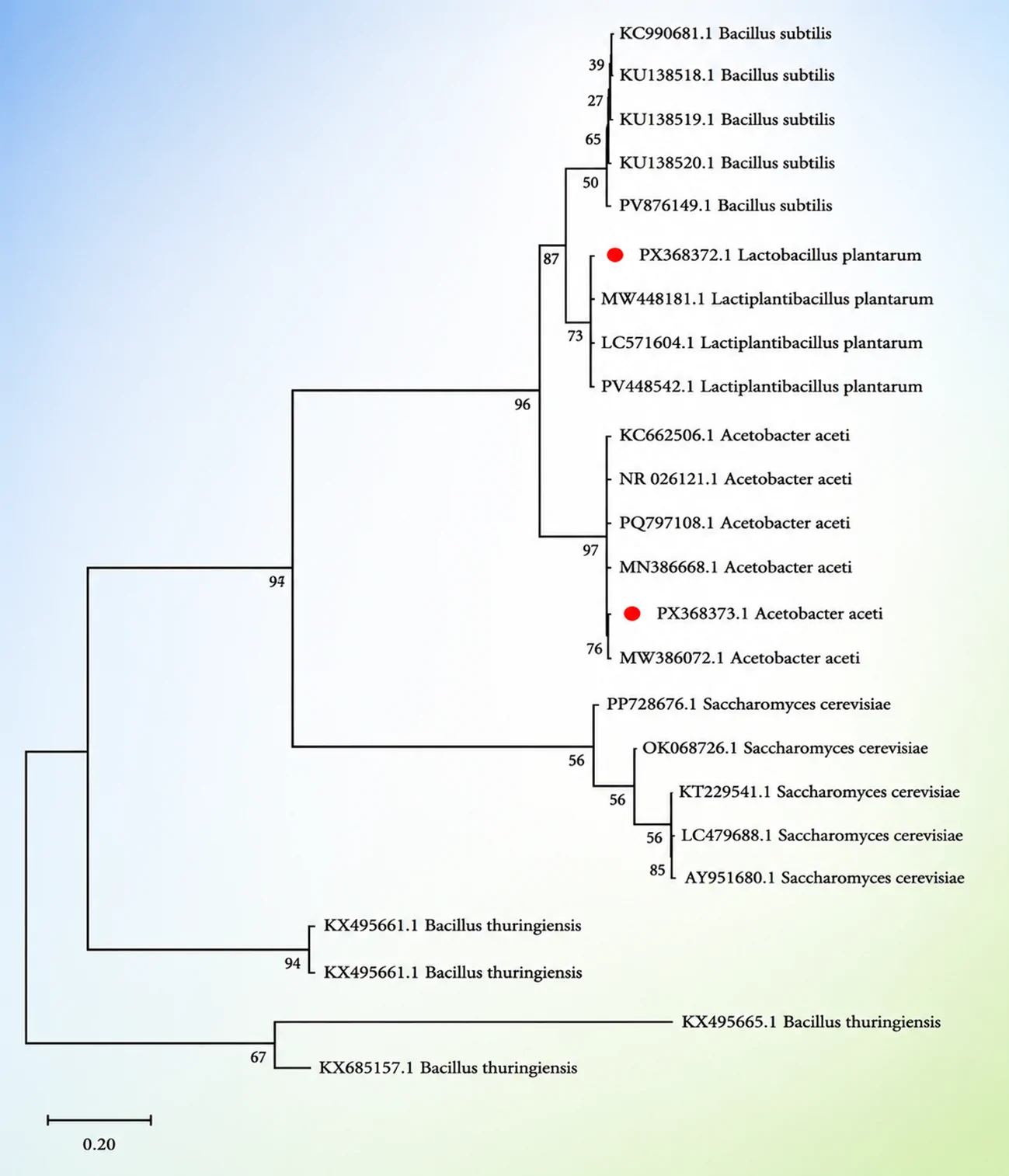
Molecular phylogenetic analysis was conducted on bacterial strains isolated from the most potent 7-day fermented banana juice. The evolutionary history was inferred using the Neighbor-Joining method. The phylogenetic tree illustrates the relationships between the query sequences (indicated by red stars) and closely related reference strains retrieved from the NCBI GenBank database. The isolates were identified as *L. plantarum* (GenBank accession: PX986372.1) and *A. aceti* (GenBank accession: PX986373.1), along with their representative reference species.

### 3.5 Phylogenetic tree construction

Phylogenetic analysis further supported the taxonomic assignment of the microbial isolates. Multiple sequence alignments were performed using closely related reference sequences, and phylogenetic trees were constructed using the Neighbor-Joining method with 1,000 bootstrap replicates. The yeast isolate was grouped within the *S. cerevisiae* clade, showing a close relationship with reference *S. cerevisiae sequences* (**Figure 7**). The bacterial isolates clustered within the *L. plantarum* and *A. aceti* clades, respectively, with strong bootstrap support (>90%), indicating reliable phylogenetic placement (**Figure 8**). The phylogenetic trees clearly separated yeast and bacterial lineages, and the isolates showed close evolutionary relationships with their corresponding reference strains based on sequence similarity. Overall, the phylogenetic analysis corroborated the ITS- and 16S rRNA gene-based identification of the isolates and confirmed their placement within established fermentative microbial groups. These identified microorganisms may contribute to fermentation-associated biochemical changes, including the generation of organic acids, alcohols, and volatile compounds, which may influence the biological properties of fermented fruit waste extracts.

### 3.6 GC-MS analysis

#### 3.6.1 Fresh banana waste fruit juice using GC-MS analysis

GC–MS analysis of fresh banana (*M. acuminata* AAA Group ‘Kluai Hom Thong’) waste fruit juice resulted in the tentative identification of 23 compounds, based on comparison of their mass spectra with those in the available spectral library (**Figure 9**; **Table 1**). Compounds with relative abundances ≥4% were tentatively identified as γ-sitosterol (10.16%), 13,27-cycloursan-3-one (7.63%), N-aminopyrrolidine (5.99%), n-hexadecanoic acid (4.59%), and 9,12-octadecadienoic acid (4.39%). Compounds with relative abundances between 2% and 4% included putative 9,12,15-octadecatrienoic acid (3.79%), thymine (2.32%), and a pyridine derivative (2.49%). Several other chromatographic peaks were assigned to esters, fatty acid derivatives, phenolic compounds, and lactone-related compounds, each accounting for less than 2% of the total relative abundance (**Figure 9**; **Table 1**). Among the tentatively identified volatile compounds, propanoic acid, 2-methyl-, hexyl ester was detected and may contribute to the volatile odor profile of banana waste juice. However, the potential impact of this compound on odor or biological activity cannot be determined solely from the GC–MS library-based assignment. Overall, the chromatographic profile included assigned phytosterols, fatty acids, nitrogen-containing compounds, esters, and other oxygenated compounds. These results provide a preliminary characterization of the chemical constituents present in fresh banana waste juice.

**Figure 9:**
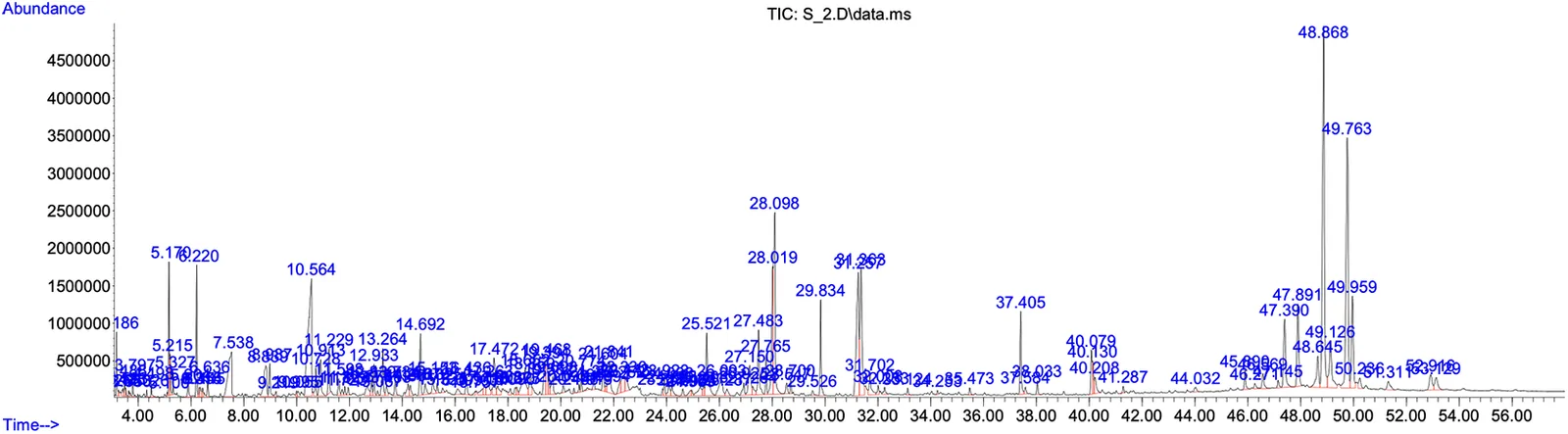
Identification of chemical constituents in fresh banana (*M. acuminata*, AAA group, “Kluai Hom Thong”) waste fruit juice using GC-MS analysis.

**Table 1:** Identification of chemical constituents from using fresh banana (*M. acuminata*, AAA Group, “Kluai Hom Thong”) waste fruit juice using GC-MS analysis.

S. no	RI (min)	Area %	Molecule name	Molecular weight	Molecule formula	Molecule structure
1	5.170	1.47	2,4-Dihydroxy-2,5-dimethyl-3(2H)-furan-3-one	128.12	C_6_H_8_O_3_	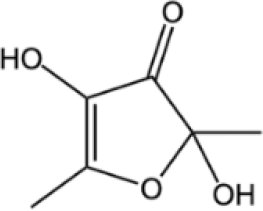
2	6.220	1.73	Ethoxymethoxyacetic acid, ethyl ester	148.16	C_6_H_12_O_4_	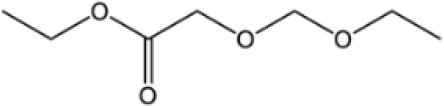
3	7.538	2.32	Thymine	126.12	C_5_H_6_N_2_O_2_	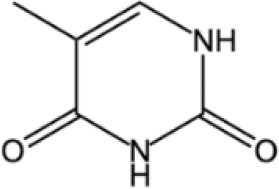
4	8.839	1.38	Propanoic acid, 2-methyl-, hexyl ester	172.26	C_10_H_20_O_2_	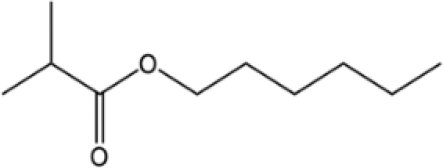
5	10.564	5.99	N-Aminopyrrolidine	86.14	C_4_H_10_N_2_	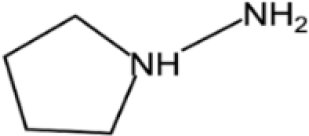
6	14.692	1.12	4-Methoxycarbonyl-4-butanolide	186.20	C_9_H_14_O_4_	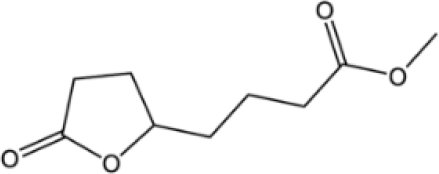
7	17.472	1.06	4-Mercaptophenol	126.17	C_6_H_6_OS	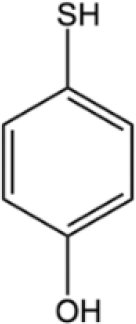
8	18.652	1.41	Benzoic acid, 4-hydroxy-	138.12	C_7_H_6_O_3_	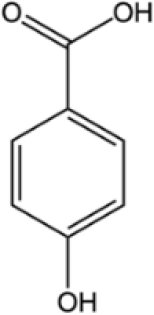
9	25.521	1.79	2,4,6-Trimethoxytoluene	182.21	C_10_H_14_O_3_	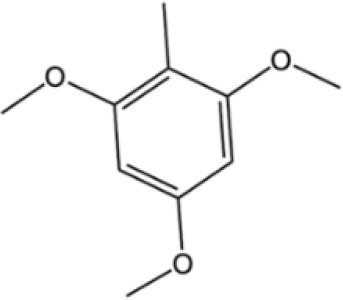
10	27.150	1.08	Succinic acid, 3,3-dimethylbut-2-yl pentyl ester	258.35	C_15_H_28_O_4_	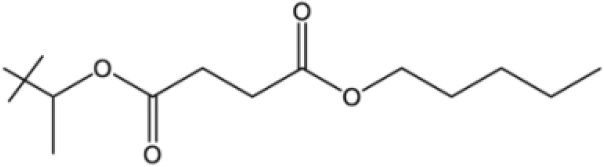
11	27.483	1.40	Aspidinol	224.25	C_12_H_16_O_4_	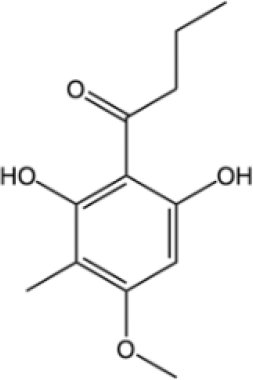
12	28.019	2.61	n-Hexadecanoic acid	256.42	C_16_H_32_O_2_	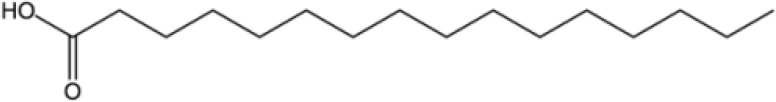
13	28.098	4.59	n-Hexadecanoic acid	256.42	C_16_H_32_O_2_	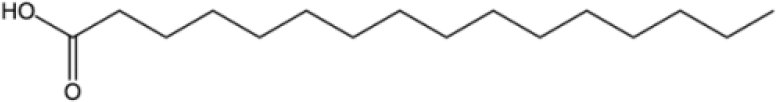
14	29.834	1.51	Benzene, 1,2,3-trimethoxy-5-(1-acetoxy-2-propenyl)-	208.25	C_12_H_16_O_5_	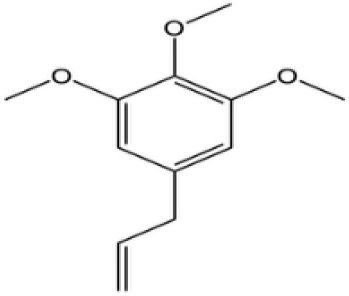
15	31.257	4.39	9,12-Octadecadienoic acid (Z,Z)-	280.44	C_18_H_32_O_2_	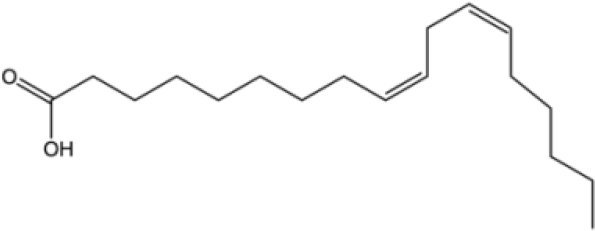
16	31.363	3.79	9,12,15-Octadecatrienoic acid, (Z, Z,Z)-	278.42	C_18_H_30_O_2_	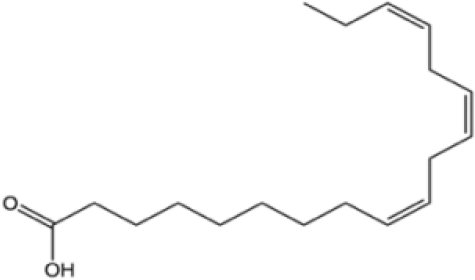
17	37.405	1.45	Hexadecanoic acid, 2-hydroxy-1-(hydroxymethyl)ethyl ester	330.49	C_19_H_38_O_4_	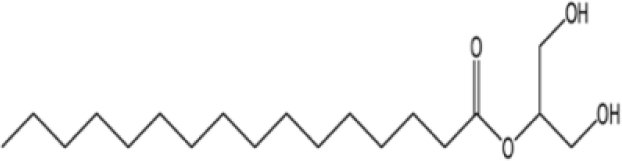
18	47.390	1.96	Campesterol	400.66	C_28_H_48_O	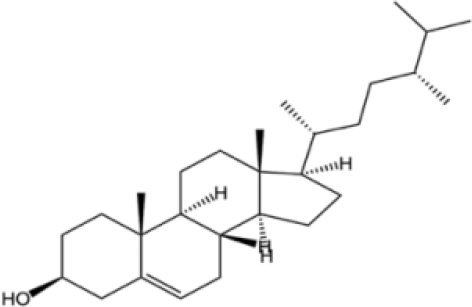
19	47.891	2.07	Stigmasterol	412.67	C_29_H_48_O	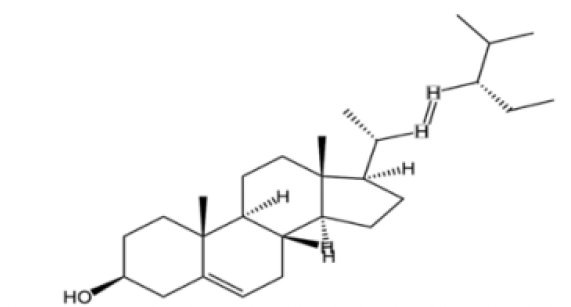
20	48.868	10.16	gamma.-Sitosterol	414.69	C_29_H_50_O	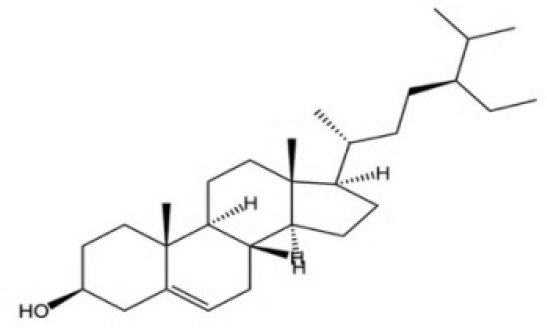
21	49.126	1.88	Stigmasta-5,24(28)-dien-3-ol, eta.,24Z)-	412.67	C_29_H_48_O	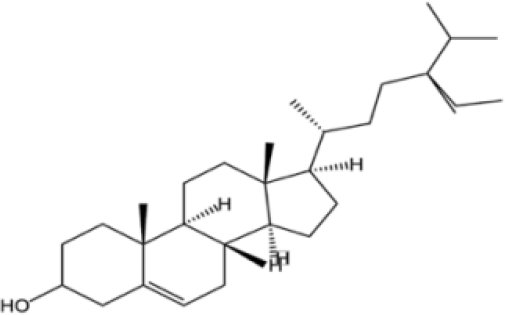
22	49.763	7.63	13,27-Cycloursan-3-one	424.68	C_30_H_48_O	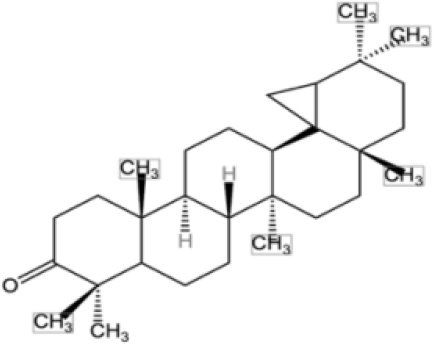
23	49.959	2.49	Pyridine, 2-ethoxy-3-[5-(4-fluorophenyl)-[1,2,4]oxadiazol-3-yl]-6-methyl-	299.30	C_16_H_14_FN_3_O_2_	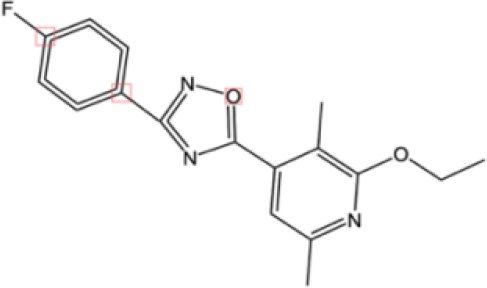

#### 3.6.2 Seven days old fermented banana waste fruit juice using GC-MS analysis

GC–MS analysis of banana (*M. acuminata* AAA Group ‘Kluai Hom Thong’) waste fruit juice fermented for 7 days resulted in the tentative identification of 27 compounds based on comparison of their mass spectra with an available spectral library, compared to 23 tentatively identified compounds in the fresh juice (**Figure 10**; **Table 2**). The greater number of compound identifications in the fermented juice indicates a more complex chromatographic profile after fermentation; however, the presence of additional compounds alone does not confirm specific biochemical transformations during fermentation. Compounds with relative abundances ≥5% were identified as a fluoren-9-one derivative (9.58%), tris(tert-butyldimethylsilyloxy)arsane (9.37% and 6.80%), triethylphosphine (6.52%), and a phosphoric acid derivative (5.81%). Other chromatographic peaks were assigned to esters, siloxane derivatives, hydrocarbons, and nitrogen-containing compounds, generally occurring at lower relative abundances (**Figure 10**; **Table 2**).

**Figure 10:**
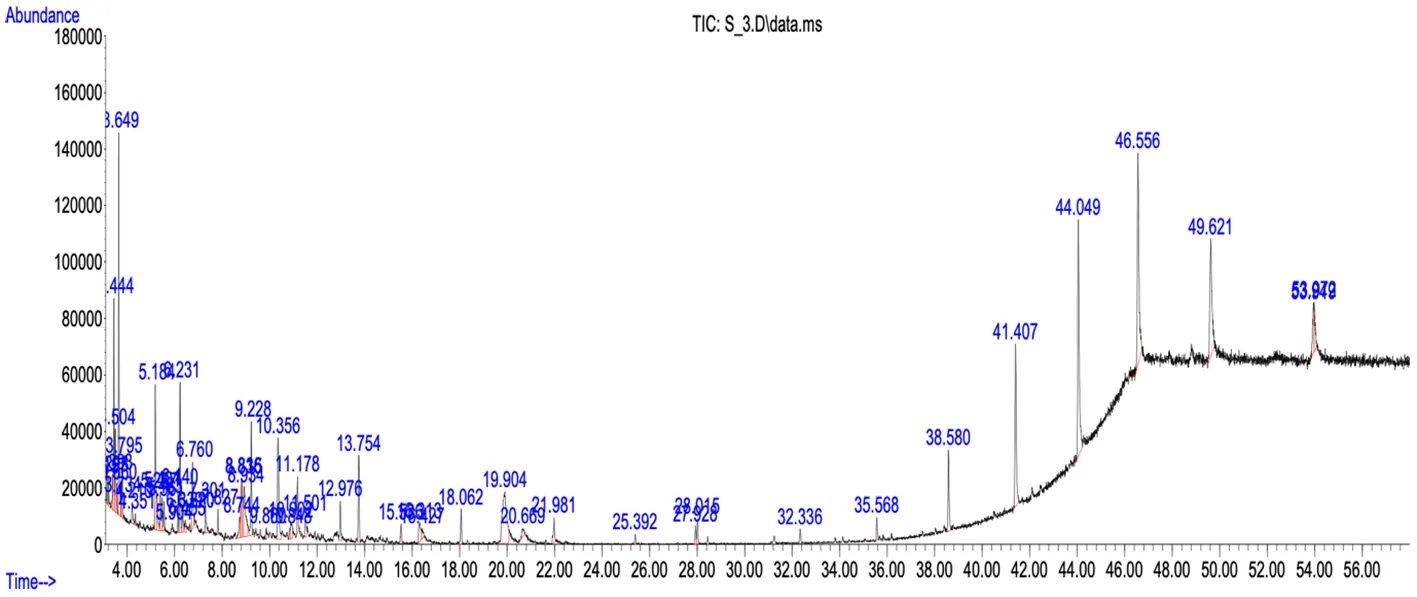
Identification of chemical constituents in potent 7-day-old fermented banana (*M. acuminata*, AAA Group, “Kluai Hom Thong”) waste fruit juice using GC-MS analysis.

**Table 2:** Identification of chemical constituents from using potent 7 days old fermented banana (*Musa acuminata*, AAA Group, “Kluai Hom Thong”) waste fruit juice using GC-MS analysis.

S. no	RI	Area %	Molecule name	Molecular weight (g/mol)	Molecule formula	Molecule structure
1	3.44	3.48	Cyclohexene, 1-ethyl-	110.1968	C_8_H_14_	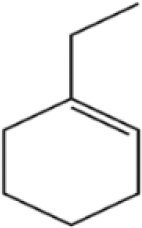
2	3.504	2.13	Acetamide, N,N’-carbonylbis-	144.13	C_5_H_8_N_2_O_3_	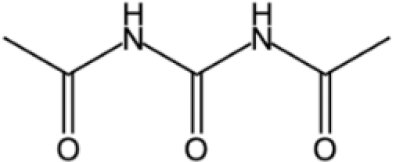
3	3.649	6.52	Triethylphosphine	118.15	C_6_H_15_P	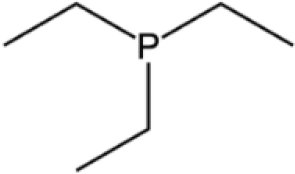
4	3.795	1.09	Nonane	128.25	C_9_H_20_	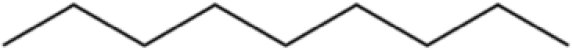
5	5.184	3.48	Phenol	94.11	C_6_H_6_O	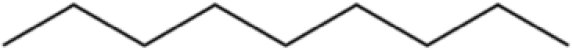
6	5.257	1.06	Ribitol	152.15	C_5_H_12_O_5_	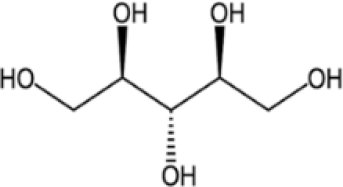
7	5.401	1.05	Hexanoic acid, 3,5,5-trimethyl-, heptyl ester	256.4241	C_16_H_32_O_2_	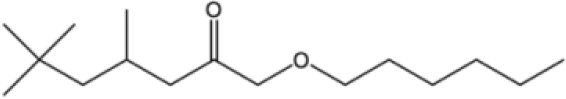
8	6.231	3.22	D-Limonene	136.23	C_10_H_16_	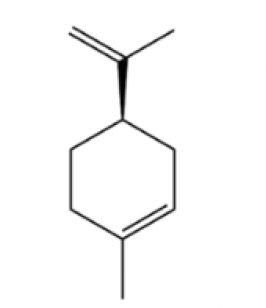
9	6.760	1.93	Trans-1-Nitro-1-propene	87.08	C_3_H_5_NO_2_	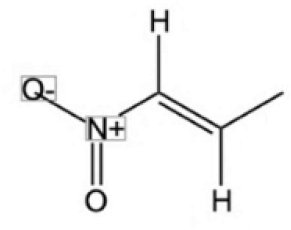
10	8.816	1.71	Hydrazine, 2-propenyl-	72.11	C_3_H_8_N_2_	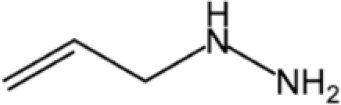
11	8.835	1.77	Propanal, 2,3-dihydroxy-, (S)-	90.08	C_3_H_6_O_3_	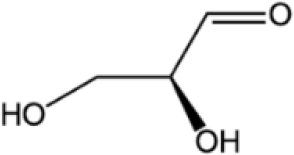
12	8.934	2.60	Sulfide, cyclopentyl isopropyl	144.28	C_8_H_16_S	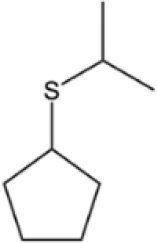
13	9.228	2.69	Cyclopentasiloxane, decamethyl-	370.79	C_10_H_30_O_5_Si_5_	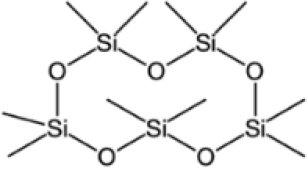
14	10.356	3.93	N-Aminopyrrolidine	86.13	C_4_H_10_N_2_	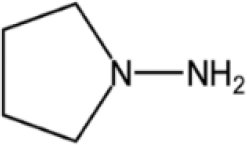
15	11.178	1.44	2-Propenoic acid, 6-methylheptyl ester	184.27	C_11_H_20_O_2_	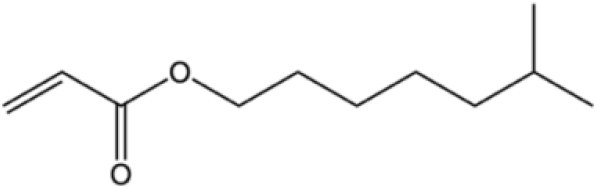
16	12.976	1.08	Tridecane	184.35	C_13_H_28_	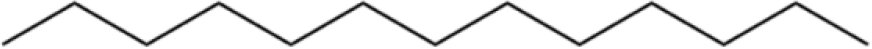
17	13.754	2.34	Cyclohexasiloxane, dodecamethyl-	444.95	C_12_H_36_O_6_Si_6_	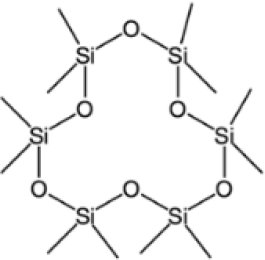
18	16.313	1.09	3-Pyrrolidinol	87.12	C_4_H_9_NO	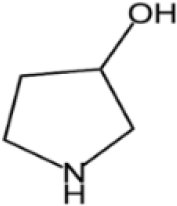
19	18.062	1.00	Cycloheptasiloxane, tetradecamethy l-	519.11	C_14_H_42_O_7_Si_7_	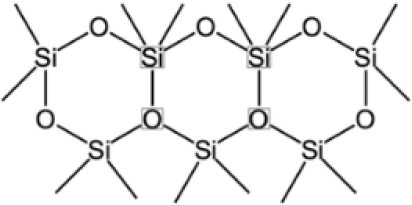
20	19.904	4.60	Ethyl 2,3-epoxybutyrate	130.14	C_6_H_10_O_3_	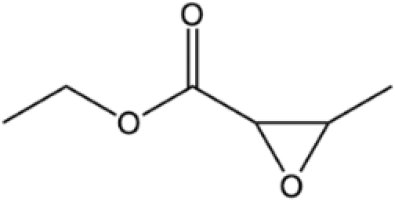
21	38.580	2.37	Pyrimidin-2(1H)-one, 6-trifluoromethyl-4-[2-(3-ethoxy-4-methoxypheny l) ethenyl]-	308.26	C_15_H_11_F_3_N_2_O_2_	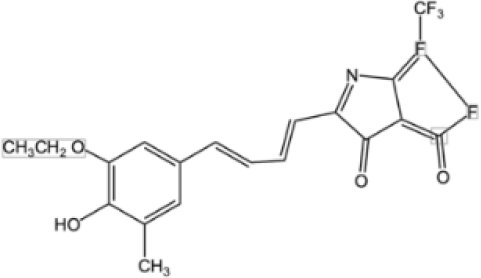
22	41.407	5.81	Phosphoric acid, 4-methylphenyl diphenyl ester	338.28	C_19_H_15_O_4_P	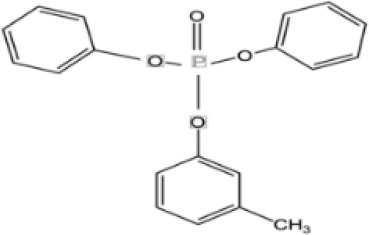
23	44.049	9.58	Fluoren-9-one, 4-(3,4-dihydro-2H-quinoline-1-carbonyl)-	337.36	C_23_H_17_NO_2_	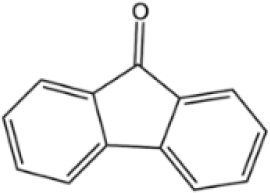
24	46.556	9.37	Tris(tert-butyldimethylsilyloxy)arsane	468.7	C_18_H_45_AsO_3_Si_3_	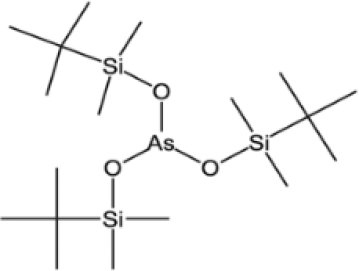
25	49.621	6.80	Tris(tert-butyldimethylsilyloxy)arsane	468.7	C_18_H_45_AsO_3_Si_3_	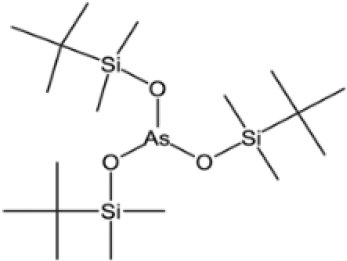
26	53.94	1.53	Acetamide, N-[4-(trimethylsilyl)phenyl]-	207.35	C_11_H_17_NOSi	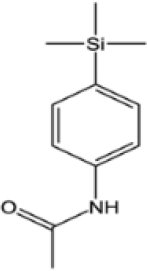
27	53.972	1.66	Methyltris(trimethylsiloxy)silane	278.70	C_10_H_30_O_3_Si_4_	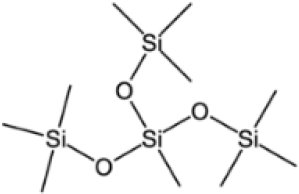

Compared to the fresh banana waste juice, the 7-day fermented juice exhibited a greater number of identified compounds and a broader range of volatile and semi-volatile compounds, as indicated by the GC–MS chromatographic profile. Several compounds, including D-limonene, phenol, ethyl 2,3-epoxybutyrate, and 2-propenoic acid, 6-methylheptyl ester, were tentatively identified in the fermented juice. These compounds may contribute to the volatile chemical profile and odor characteristics of the formulation; however, their individual effects on odor or attraction of *D. melanogaster* cannot be determined from GC–MS profiling alone.

The stronger attraction response observed for the 7-day fermented juice in the behavioral bioassays coincided with differences in its GC–MS profile compared to the fresh juice. This correlation suggests that variations in the chemical composition of the fermented formulation may contribute to its attractant properties, although the current GC–MS data do not establish a direct causal link between individual compounds and fly attraction. Overall, the 7-day fermented banana waste juice exhibited a more complex chromatographic profile than the fresh juice, featuring several identified volatile and semi-volatile compounds. These findings provide preliminary chemical evidence of differences between fresh and fermented banana waste juice and support further investigation into the specific compounds responsible for the observed attraction of *D. melanogaster*.

### 3.7 Greenhouse insect attractant bioassay

Following laboratory screening, in which 7-day fermented banana waste juice elicited the highest attraction response among the fruit-waste formulations tested, this formulation was selected for subsequent greenhouse evaluation. The greenhouse attractant bioassay revealed significant differences in the attraction responses of adult *D. melanogaster* among the tested attractant formulations. Sodium alginate–CMC beads containing 7-day-fermented banana waste juice exhibited the highest attraction response (82.96%; F_(2,6)_ = 358.183, p < 0.001), followed by beads containing fresh (0-day) banana juice (**Figure 11**). These results demonstrate that the 7-day-fermented banana juice retained strong attractant activity following encapsulation in sodium alginate–CMC beads. The enhanced attraction observed with the fermented formulation suggests that the bead-based delivery system may provide an effective means of releasing fermentation-associated volatile compounds and could therefore have potential as a controlled-release attractant formulation for *D. melanogaster* management.

**Figure 11:**
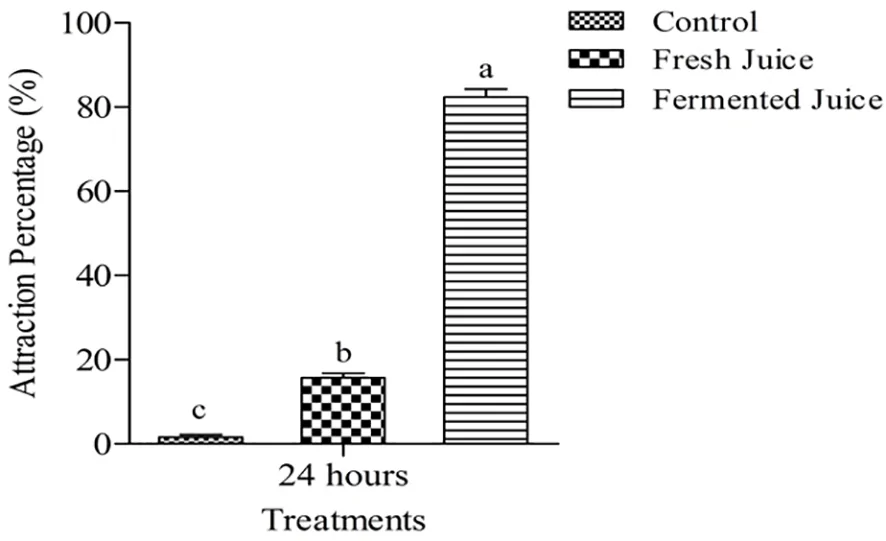
Attraction response of *D. melanogaster* to more potent banana fruit waste-derived juices at 0 and 7 days of fermentation, compared to a control, under greenhouse conditions.

### 3.8 Binding pocket prediction and molecular docking analysis of candidate semiochemicals with dmelOBP28a

#### 3.8.1 Molecular docking of 2-propenoic acid, 6-methylheptyl ester with dmelOBP28a

Cavity detection on the odorant-binding protein dmelOBP28a from *D. melanogaster* (PDB: 6QQ4) identified five candidate binding pockets (C1–C5), which were evaluated as docking sites for 2-propenoic acid, 6-methylheptyl ester (PubChem CID 165746). The predicted pockets differed in cavity volume, spatial location, and docking (Vina) score (**Figure 3**; **Table 3**). Pocket C1 produced the most favorable Vina score of −6.7 kcal/mol, the most favorable predicted docking score recorded among all pockets and ligands assessed in this study. C1 outperformed the second-best pocket (C5, −5.9 kcal/mol) by 0.8 kcal/mol, despite C5 possessing a considerably smaller cavity (335 Å³ versus 1468 Å³) (**Figure 3**; **Table 3**). The convergence of the most favorable binding energy with the largest cavity volume (2.7–4.3-fold greater than the other pockets) indicates that C1 constitutes the principal ligand-binding site of dmelOBP28a for this ligand, consistent with the deep, elongated hydrophobic cavity characteristic of insect OBPs that accommodate long-chain lipophilic molecules such as branched fatty acid esters.

**Table 3:** Docking scores and cavity characteristics of 2-Propenoic acid, 6-methylheptyl ester, D-Limonene, Ethyl 2,3-epoxybutyrate with Odorant-binding protein dmelOBP28a from *D. melanogaster* (PDB ID: 6QQ4).

The ligand	Vina score
2-Propenoic acid, 6-methylheptyl ester	-6.7
D-Limonene	-6.5
Ethyl 2,3-epoxybutyrate	-5.0

The C1 pocket is delineated by 35 residues, all contributed by chain A — the largest residue set observed across all predicted pockets — comprising an extensive hydrophobic/aliphatic surface (Leu7, Leu10, Met31, Met50, Ile55, Leu62, Ala66, Ala71, Tyr74, Ala85, Leu86, Ile88, Phe112, Ala116, Leu121, Leu122) capable of accommodating the branched 6-methylheptyl chain of the ester, together with polar and charged residues positioned at the pocket entrance (Glu3, Glu30, Lys34, Gln35, Arg46, Asp63, Glu65, His68, Lys70, Asp90, Glu106, Arg113) likely engaging the acrylate carbonyl head group (**Figure 3**, **Table 3**). The second-ranked pocket, C5 (−5.9 kcal/mol), is also located exclusively within chain A and shares numerous residues with C1 (Leu7, Leu10, Met31, Lys34, Gln35, Arg46, Val49, Met50, Lys70, Ala71, Tyr74, Thr75, Ala85, Leu86, Ile88, Gly89, Phe112, Arg113, Ala116, Leu121, Leu122), suggesting that C1 and C5 represent overlapping or adjoining regions of a single extended hydrophobic groove rather than two independent cavities. The remaining pockets (C2–C4) exhibited comparatively weaker docking scores (−4.4 to −5.0 kcal/mol) and smaller cavity volumes; C4, and likely C3, involved residues from both chains A and B, indicating that these correspond to interfacial or surface-exposed sites rather than the primary internal binding cavity.

#### 3.8.2 Molecular docking of D-Limonene with dmelOBP28a

Docking of D-limonene (PubChem CID 440917) against the five predicted cavities of dmelOBP28a (**Figure 4**; **Table 3**) similarly identified pocket C1 as the most favorable binding site, with a Vina score of −6.5 kcal/mol — substantially stronger than all remaining pockets, exceeding the next-best site (C4) by ≥1.3 kcal/mol. As with the previous ligand, C1 possessed the largest cavity volume (1468 Å³), 2.5- to 4-fold greater than the other pockets, reinforcing its identification as the principal ligand-binding site and consistent with the deep hydrophobic cavity typical of insect OBPs that bind volatile monoterpenes.

The C1 pocket comprises 24 residues, entirely from chain A, predominantly hydrophobic and aliphatic in character (Leu7, Leu10, Met31, Met50, Ile55, Ala71, Tyr74, Ala85, Leu86, Ile88, Phe112, Ala116, Leu121, Leu122), consistent with a nonpolar binding environment well suited to the hydrocarbon skeleton of D-limonene. A smaller subset of polar and charged residues (Glu3, Lys34, Lys70, Gln73, Thr64, Thr75, Arg113) lines the pocket periphery, likely contributing to entrance solvation rather than direct ligand contact. The remaining pockets (C2–C5) yielded weaker docking scores (−3.8 to −5.2 kcal/mol) and smaller cavity volumes, with C3 and C4 involving residues from both chains A and B, suggesting interfacial rather than primary ligand-binding character. Notably, D-limonene exhibited a markedly stronger predicted affinity for pocket C1 at the same site, consistent with its larger hydrophobic surface area enabling more extensive van der Waals contacts within the cavity.

#### 3.8.3 Molecular docking of ethyl 2,3-Epoxybutyrate with dmelOBP28a

Docking of ethyl 2,3-epoxybutyrate (PubChem CID 29774) identified pocket C1 as the most favorable binding site (Vina score = −5.0 kcal/mol), closely followed by C2 (−4.9 kcal/mol) (**Figure 5**; **Table 3**). C1 again possessed by far the largest cavity volume (1468 Å³), 1.5- to 4-fold greater than the remaining pockets, whereas C2, despite its comparable score, was substantially smaller (939 Å³) and located entirely within chain B. The concurrence of the top docking score with the largest cavity volume supports C1 as the most probable physiological binding site for this ligand. The C1 pocket is lined by 28 residues, all from chain A, comprising hydrophobic/aliphatic residues (Leu7, Leu10, Met31, Val49, Met50, Ile55, Ala71, Tyr74, Ala85, Leu86, Ile88, Phe112, Ala116, Leu121, Leu122) alongside polar and charged residues (Lys34, Gln35, Arg46, Asp63, Lys70, Asp90, Glu106, Arg113) distributed around the cavity. This residue composition suggests the hydrophobic ethyl and methyl substituents of the ligand are accommodated by the aliphatic residues, while the polar epoxide and ester moieties may engage the surrounding charged residues.

The remaining pockets (C2–C5) exhibited comparable-to-weaker docking scores (−3.5 to −4.9 kcal/mol) with smaller cavity volumes; C3 and C4 involved residues from both chains, indicating interfacial or surface-exposed character rather than the primary internal binding site.

## 4 Discussion

Fruit waste was selected as the primary substrate due to its high availability, low cost, nutritional richness, and strong potential for microbial fermentation and volatile compound production. Fruit-processing and market activities generate substantial quantities of discarded fruit residues, which are rich in readily fermentable carbohydrates, sugars, organic acids, and other nutrients that support the growth and metabolic activity of yeasts and bacteria. These characteristics make fruit waste particularly suitable for generating fermentation-associated metabolites and volatile organic compounds (VOCs) that can influence insect olfactory behavior. The choice of fruit waste was therefore motivated not only by its abundance and potential for waste valorization but also by its chemical and biological suitability for producing ecologically relevant odor cues. This rationale is especially pertinent to *D. melanogaster*, which is closely associated with ripe, damaged, and fermenting fruits and relies on microorganism- and fermentation-derived volatiles to locate suitable feeding and reproductive resources. Thus, biotransformation of fruit waste through controlled fermentation offers a biologically relevant approach to enhance its attractiveness while simultaneously supporting circular economy principles by converting an underutilized waste stream into a value-added insect attractant.

In the present study, fermentation duration significantly influenced the attractiveness of fruit-waste-derived juice formulations to adult *D. melanogaster*, with attraction generally increasing with fermentation time. Among the substrates evaluated, 7-day-fermented banana (*M. acuminata* AAA Group ‘Kluai Hom Thong’) waste juice elicited the highest attraction response (**Figures 6**, **11**). Similarly, Cong et al. (36) reported that Actinidia chinensis exhibited the strongest attractiveness to *D. melanogaster* among the four fruits tested. Their subsequent analyses identified m-cymene as a concentration-dependent semiochemical, attracting the flies at concentrations of 0.2–0.7% but repelling them at 1.0%. This finding highlights the importance of both volatile composition and concentration in determining the behavioral responses of *D. melanogaster*. Consistent with the present study, Zhu et al. (37) found that odors from overripe mangoes were more attractive to adult *D. melanogaster* than those from overripe plums, pears, and grapes. GC–EAD analyses identified ethanol, acetic acid, amyl acetate, 2-phenylethanol, and phenylethyl acetate as antennally active volatiles, while a blend of ethanol, acetic acid, and 2-phenylethanol elicited stronger attraction than individual compounds. These findings underscore the importance of fruit-derived volatile blends in mediating *D. melanogaster* attraction. This present study suggests that fermentation modified the chemical characteristics of the fruit substrate and may have enhanced the production or release of volatile cues attractive to *D. melanogaster*. The strong response to fermented banana waste juice is consistent with the ecological association of *D. melanogaster* with fermenting fruit and microorganisms. Yeast-associated fermentation products, including ethanol, acetic acid, acetoin, higher alcohols, and esters, can function as olfactory cues that help drosophilid flies locate nutrient-rich microbial substrates (38–40). The attractiveness of fermented banana juice compared with the other fruit substrates may reflect differences in substrate composition and microbial fermentation. Banana contains readily fermentable carbohydrates that can support microbial growth and metabolite production.

During fermentation, microbial metabolism can alter the relative abundance of alcohols, organic acids, esters, aldehydes, ketones, and other volatile compounds, thereby modifying the overall odor profile of the substrate (10, 40). Thus, the enhanced attractiveness of fermented banana juice may be associated with differences in the composition and relative abundance of fermentation-derived volatiles rather than a single attractant compound. The ecological relevance of fermentation-associated odors has also been demonstrated in other drosophilid species. *Drosophila suzukii*, for example, responds to combinations of fruit- and fermentation-associated volatiles, although its host-use ecology differs from that of *D. melanogaster*. Whereas *D. melanogaster* commonly exploits damaged, overripe, and fermenting fruit, *D. suzukii* can oviposit in intact ripening soft-skinned fruit using its serrated ovipositor. Therefore, the relative importance of fermentation- and plant-derived odor cues may differ between these species (41, 42). This ecological distinction further supports the interpretation that the strong attraction observed in the present study is consistent with the established association of *D. melanogaster* with fermenting fruit and microbe-rich resources.

The isolation of *S. cerevisiae*, *L. plantarum*, and *A. aceti* from the selected fermented banana juice provides further evidence that microbial activity contributed to the fermentation process. Yeasts are important contributors to alcoholic fermentation and can produce ethanol and numerous volatile metabolites, whereas lactic acid bacteria and acetic acid bacteria can modify organic acid concentrations and other physicochemical properties of the substrate (43). The presence of these microorganisms may therefore have contributed to the progressive changes in the odor profile observed during fermentation. However, because the present study did not experimentally manipulate individual microbial species, the specific contribution of each isolate to attractant production cannot be established. Future studies using defined microbial cultures would be required to determine the individual and combined effects of these microorganisms on VOC production and fly attraction.

The GC–MS analysis further indicated that fermentation was associated with substantial changes in the volatile and semi-volatile chemical profile of banana waste juice (**Figures 10**, **2**; **Tables 1**, **2**). The fermented sample contained a range of alcohols, esters, terpenoid compounds, phenolic compounds, and other volatile constituents (**Tables 1**, **2**). Such complex mixtures are ecologically relevant because insect olfactory systems respond to combinations of chemical cues rather than necessarily to individual compounds. Accordingly, the attraction observed in the present study is likely to reflect the combined effects of multiple fermentation-associated compounds. Consistent with the present findings, *D. melanogaster* detects fermentation-associated esters, including ethyl hexanoate and ethyl octanoate, through the odorant receptors Or22a and Or22b. Natural variation at the Or22 locus influences preference for *Saccharomyces cerevisiae* cultures that produce these esters, highlighting the ecological importance of olfactory adaptation in locating fermenting fruit resources (44). Previous studies reported by Yoon (45) on *D. suzukii* showed that fruit-derived chemicals can elicit distinct behavioral responses depending on their chemical identity. While some volatiles act as attractants, 5-hydroxymethylfurfural (5-HMF) disrupts oviposition and induces dose-dependent physiological effects, whereas palmitic and stearic acids function as short-range, contact-dependent cues. These findings highlight the importance of chemical composition and concentration in determining insect responses and support the identification of key volatile organic compounds (VOCs) underlying the attraction observed in the present study.

The development of sodium alginate–CMC beads provided a practical formulation approach for delivering the selected fermented banana juice. Alginate is widely used as an encapsulation matrix because it forms hydrogels through calcium-mediated ionic crosslinking, while incorporation of CMC can modify the physical properties and integrity of the polymer matrix (46, 47). In the present study, the fermented banana juice-loaded beads produced a substantially greater attraction response than the fresh-juice formulation and the control under greenhouse conditions (**Figure 2**). Similarly, de Souza et al. (48) developed chitosan–sodium alginate nanocapsules using alternating-layer adsorption to modulate the release of essential oils derived from three Piperaceae species—*Piper aduncum*, *Piper gaudichaudianum*, and *Piper marginatum*—for the management of *Drosophila suzukii*. This study further demonstrates the potential of biopolymer-based encapsulation systems to regulate the release of volatile compounds and enhance the stability and delivery of botanical attractants or insecticidal agents, supporting the application of biodegradable polymer-based formulations in sustainable Drosophila management. This present study demonstrates that the fermented attractant retained its behavioral activity after incorporation into the polymer matrix. Although the present experiment did not directly quantify release kinetics or volatile retention, the results indicate that sodium alginate–CMC beads have potential as a delivery system for fermented fruit-derived attractants. Further studies measuring release rates, formulation stability, field persistence, and attraction over extended periods are required to confirm the controlled-release properties of the formulation.

Molecular docking provided complementary computational evidence regarding the potential interactions between selected volatile organic compounds (VOCs) and dmelOBP28a, an odorant-binding protein (OBP) of *D. melanogaster*. The experimentally determined structure of dmelOBP28a (PDB ID: 6QQ4) was obtained by X-ray crystallography at approximately 2.0 Å resolution. Previous biochemical and genetic studies have indicated that dmelOBP28a binds a restricted group of odorants, including β-ionone, and that deletion of Obp28a can alter behavioral responses to β-ionone, supporting its involvement in specific aspects of odor detection (49). In the present study, D-limonene and selected 2-propenoic acid ester derivatives showed favorable predicted docking scores and formed interactions with residues located within the predicted dmelOBP28a binding cavity (**Figures 3**–**5**; **Table 3**). These results provide a structural hypothesis for potential interactions between VOCs present in the fermented banana formulation and dmelOBP28a. Consistent with the present findings, Li et al. (50) reported that molecular docking identified ethyl linoleate as a favorable ligand for three odorant-binding proteins (OBPs), with Lush exhibiting the strongest predicted interaction. Their results further suggested that these OBP–ligand interactions may play a role in recognizing attractant-associated compounds in rotten fruits, offering valuable structural insights for the identification and rational design of novel insect attractants.

However, because the behavioral assays evaluated complex fruit-juice formulations rather than purified compounds, the present findings cannot establish that D-limonene or any individual ester directly caused the observed attraction. Instead, these compounds should be considered candidate attractant constituents for further experimental investigation. The docking results provide valuable hypothesis-generating insights into the potential interactions between the candidate VOCs and dmelOBP28a. The favorable docking scores and predicted interactions offer a useful structural basis for prioritizing candidate compounds for further experimental investigation. Although docking scores represent computational predictions rather than experimentally measured binding affinities or biological activity, these findings provide a valuable foundation for subsequent biochemical, electrophysiological, and behavioral validation. Overall, the behavioral, microbiological, chemical, formulation, and computational results collectively demonstrate the potential of fermented banana waste as a source of *D. melanogaster* attractant material. The increase in attraction during fermentation, identification of fermentation-associated microorganisms, changes in the VOC profile, and strong behavioral response to the fermented formulation indicate that microbial fermentation can enhance the biological attractiveness of fruit waste. Furthermore, incorporation of the selected fermented juice into sodium alginate–carboxymethyl cellulose (CMC) beads demonstrated the feasibility of developing a biodegradable formulation for controlled attractant delivery.

The biological activity of individual VOCs and their interactions with dmelOBP28a were not experimentally validated, and the optimized formulation was evaluated only under controlled greenhouse conditions. Its performance may differ under field conditions because of variations in temperature, humidity, airflow, and competing environmental odors. Future research should therefore focus on (i) electrophysiological and behavioral validation of candidate VOCs using EAG and olfactometer assays, (ii) optimization of VOC combinations and bead formulations to improve release characteristics and attractant longevity, and (iii) multi-site field trials to evaluate efficacy, environmental stability, and practical applicability within integrated pest management (IPM) programs.

## 5 Conclusion

This study demonstrates that microbial fermentation significantly enhanced the attractant potential of fruit-waste-derived extracts against *D. melanogaster* under the experimental conditions evaluated. Among the tested treatments, banana (*M. acuminata* AAA Group ‘Kluai Hom Thong’) waste juice fermented for seven days showed the highest attraction, highlighting the importance of both substrate type and fermentation duration in influencing attractant activity. The identification of *S. cerevisiae*, *L. plantarum*, and *A. aceti*, together with GC–MS analysis, suggests that microbial fermentation contributed to the production of volatile compounds potentially involved in the behavioral response of *D. melanogaster*. Encapsulation of the fermented extract in sodium alginate–CMC beads improved its stability and sustained attractant performance under greenhouse conditions, suggesting its potential as a delivery system for attractant-based management approaches. Molecular docking further identified potential interactions between selected VOCs and dmelOBP28a; however, these findings require biochemical and electrophysiological validation.

Overall, this study demonstrates the potential of fruit-waste-derived fermented extracts as low-cost and potentially sustainable attractants for *D. melanogaster*. However, the present study evaluated insect attraction rather than population suppression, fruit protection, or reductions in fruit infestation and postharvest losses. Therefore, further research, including open-field validation, formulation optimization, and experimental confirmation of the roles of key VOCs in insect attraction, is needed to determine the applicability of these attractants in practical fruit-fly management and their potential contribution to reducing fruit damage and associated postharvest losses.

## Data Availability

The raw data supporting the conclusions of this article will be made available by the authors, without undue reservation.
